# *STX1B* variant-specific synaptic dysfunction is associated with network hyperexcitability in human iPSC-derived neurons

**DOI:** 10.1016/j.ebiom.2026.106451

**Published:** 2026-08-31

**Authors:** Carolin Haag, Felix Gsell, Oleg Vinogradov, Morgana Barroso Oquendo, Yuanyuan Liu, Betül Uysal, Heidi Löffler, Fabienne Stehle, Fabian Klopfer, Jan Crönlein, Kaja Böttcher, Ruud F. Toonen, Hiltrud Muhle, Bernhard Kohl, Holger Lerche, Niklas Schwarz

**Affiliations:** a Department of Neurology and Epileptology, Hertie-Institute for Clinical Brain Research, University of Tübingen, Tübingen, Germany; b Quantitative Biology Center (QBiC), University of Tübingen, Tübingen, Germany; c Department of Functional Genomics, Center for Neurogenomics and Cognitive Research, Vrije Universiteit Amsterdam, Amsterdam, the Netherlands; d Department of Neuropediatrics, University Medical Center Schleswig-Holstein, Kiel, Germany; e Department of Neuropediatrics, Hamburg Epilepsy Center, Catholic Children's Hospital Wilhelmstift, Hamburg, Germany

**Keywords:** *STX1B*, Epilepsy, Induced pluripotent stem cells, Disease modelling, Synaptic transmission

## Abstract

**Background:**

Variants in *STX1B*/syntaxin-1B are linked to a spectrum of fever-associated epilepsy syndromes. While studies in murine models have provided mechanistic insights, their relevance to human disease in a heterozygous context may be limited.

**Methods:**

We investigated two pathogenic *STX1B* variants using isolated single neurons and neuronal network cultures derived from patient-specific induced pluripotent stem cells. These carried either a *de novo* p.G226R variant, associated with severe developmental epilepsy, or an InDel variant (p.K45delinsRCMIE/p.L46M) linked to a transient familial seizure syndrome. Synaptic function and network excitability were assessed using patch-clamp and multi-electrode array recordings, alongside morphological and transcriptomic profiling.

**Findings:**

G226R exhibited both gain- and loss-of-function characteristics, with increased miniature excitatory postsynaptic current frequency in networks but not in autapses, and synaptic failure during sustained high-frequency stimulation. For the InDel variant, the predicted loss-of-function phenotype based on reduced syntaxin-1B levels was not detectable at the single-cell level, likely masked by compensatory synaptic upregulation. At the network level, however, both variants were associated with neuronal hyperexcitability, characterised by more frequent and prolonged bursting activity, with a much stronger phenotype in G226R-containing networks. Transcriptomic profiling revealed a differential dysregulation of synaptic and other neuronal genes.

**Interpretation:**

The divergence between morphological, electrophysiological and transcriptomic findings suggests that compensatory mechanisms may contribute to network hyperexcitability. Initially engaged to maintain homoeostasis, they may ultimately contribute to a pathological network state. The graded severity of network alterations across *STX1B* variants correlates with the clinical phenotypes.

**Funding:**

BMBF (Treat ION-01GM2210A, SNAREopathies-01EW1809A), 2023 FEBS Summer Fellowship, Fortüne programme (2610-0-0), EKFS college precise.net, Open Access Publishing Fund of University of Tübingen.

**Research in context**

**Evidence before this study**

Heterozygous variants in the *STX1B* gene have been associated with a wide range of epilepsy syndromes, but their functional consequences remain unclear. Previous studies using mouse neurons identified variant-specific effects only in a *Stx1a/Stx1b* double knockout background, a condition with limited relevance to human physiology, whereas functional effects were absent in a heterozygous *Stx1b* background.

**Added value of this study**

We examined two paradigmatic *STX1B* variants in a human model using patient-derived iPSC neurons. By integrating single-cell electrophysiology, network-level recordings, and molecular profiling, we uncovered distinct forms of synaptic dysfunction, covering both gain- and loss-of-function effects, that converge on a shared, but graded, network-level hyperactivity phenotype. The human iPSC model captured functional consequences not evident in heterozygous mouse systems, offering mechanistic insight into how *STX1B* variants are associated with pathological network activity in epilepsy.

**Implications of all the available evidence**

This study underscores the value of patient-specific models in unravelling the pathomechanisms of human neurological disorders. In addition, the inherent limitations of *in vitro* systems highlight the need for more advanced models that capture synaptic heterogeneity and cell-type–specific features to more precisely characterise synaptic dysfunction. Taken together, these approaches will help to further elucidate the exact mechanisms of *STX1B*-related pathology and enable the rational development of future treatment strategies.

## Introduction

Neuronal communication relies on the conversion of electrical signals into chemical ones, facilitated by the controlled release of neurotransmitter-filled vesicles at presynaptic sites. This release is primarily mediated by the three neuronal N-ethylmaleimide-sensitive factor attachment protein receptor (SNARE) proteins: syntaxin-1, synaptobrevin, and SNAP-25.^1^ The assembly of this core SNARE complex drives the fusion of the vesicle membrane with the presynaptic membrane, releasing enough energy to overcome the energetic barrier of fusing two negatively charged phospholipid bilayers.^2^^,^^3^

Through its interactions with other SNARE-associated proteins, syntaxin-1 emerges as a key player in regulating the fusion process.^4^, ^5^, ^6^, ^7^ This regulatory function is driven by the presence of a distinctive N-terminal H_abc_-domain, which is structurally complemented by the SNARE-specific subdomain that is essential for the complex assembly.^8^^,^^9^ The significance of these two subdomains is also highlighted by their conservation among the two neuronal isoforms, syntaxin-1a and syntaxin-1b, which exhibit 84% homology in their amino acid sequences.^10^ This structural similarity suggests partial functional redundancy. Indeed, while knockout of one of the two other SNARE proteins, synaptobrevin and SNAP-25, results in a marked reduction in evoked synaptic transmission,^11^^,^^12^ *Stx1a*^−/−^ mice develop normally and show no major effect on fast synaptic transmission.^13^ In contrast, *Stx1b*^−/−^ mice die shortly after birth and exhibit synaptic defects such as a reduced frequency of miniature postsynaptic currents, highlighting the distinct and more critical role of *Stx1b* in maintaining basal synaptic function.^14^, ^15^, ^16^

The importance of the human *STX1B* gene was further highlighted in a study by our group, which associated variants in *STX1B* with a wide spectrum of genetic epilepsy syndromes.^17^ Since then, *STX1B*-associated epilepsies form a distinct subgroup within the broader category of disorders related to synaptic dysfunction, collectively known as synaptopathies.^18^ The pathogenic variants in *STX1B* extend across the entire protein structure, but cluster particularly in the regulatory H_abc_-domain as well as the SNARE motif.^19^ *STX1B*-related synaptopathies present with clinical manifestations that vary in severity from mild genetic epilepsy with febrile seizures plus (GEFS+) to severe forms of developmental and epileptic encephalopathies (DEE), with the latter exhibiting severe seizures and concurrent developmental delay or regression.^19^ The causal pathogenic variants are equally diverse and encompass missense or truncating variants as well as partial or even whole gene deletions. Our group could show that protein truncating variants were mostly associated with milder syndromes, whereas missense variants particularly in the SNARE motif caused more often severe encephalopathies.^19^ Even though all these variants affect the function of the same protein, this variant diversity appears to give rise to various synaptic dysfunctions, ultimately matching the clinical heterogeneity.

In an attempt to observe the proposed diversity of synaptic malfunctions at the molecular level, a previous study by Vardar et al. investigated three paradigmatic *STX1B* variants V216E, G226R, and InDel (p.K45delinsRCMIE and p.L46M) using patch-clamp electrophysiology on mouse autaptic cultures, and biochemical and structural approaches.^20^ This led to the identification of distinct synaptic dysfunctions for all three variants when expressed on a *Stx1a/Stx1b* double knockout background. Specifically, the InDel variant resulted in an unfolded syntaxin-1B protein that completely failed to sustain neurotransmission, whereas G226R displayed partial loss-of-function (LOF) characteristics. V216E, in contrast, displayed gain-of-function (GOF) features, evident as an increased release probability of synaptic vesicles. Notably, the synaptic malfunctions were mostly absent in a heterozygous *Stx1b*^+/−^ background. As this approach aimed to more closely mimic the heterozygous patient situation, the translatability of the synaptic phenotypes in a double knockout condition in mice remains under debate.

To overcome this limitation, we investigated two of these pathogenic *STX1B* variants in a human-relevant context. We used patient-specific induced pluripotent stem cell (iPSC)-derived neurons carrying either the aforementioned G226R missense variant or the complex InDel variant. While the investigation at the single-cell autaptic level allowed an in-depth analysis of synaptic electrophysiology, the concurrent analysis using multi-electrode arrays (MEAs) demonstrated the network consequences of the synaptic dysfunction. Finally, morphological and transcriptomic analyses further suggested the involvement of secondary mechanisms that may connect the primary synaptic deficits to the observed hyperexcitable network state. Understanding both the phenotypic diversity and the shared features among *STX1B*-related synaptopathies is crucial for advancing the development of potentially tailored therapeutic strategies.

## Methods

### Generation and validation of iPSC lines

The two carriers of the InDel variant were both female and related (mother and daughter). Both individuals were diagnosed with GEFS+ and are currently seizure-free without medication. Skin biopsies were obtained in October 2016, at the ages of 36 and 56 years, respectively. The patient carrying the G226R variant was also female, and the skin biopsy was collected in May 2020, at the age of 14 years. This patient was diagnosed with DEE and is under seizure control with levetiracetam, stiripentol, and valproate. The two control individuals were unrelated, healthy, non-commercial donors. iPSCs from Control 1 were derived from skin fibroblasts obtained from foreskin tissue collected during a circumcision in February 2013 at the age of 6 years from a male donor, while cells from Control 2 were derived from keratinocytes isolated from a hair root of a female donor in October 2014 at the age of 30 years. All donors were of Caucasian ancestry. The two control lines were reprogrammed in 2013 and 2014 and were maintained as cryopreserved iPSCs until use, whereas all patient-derived lines were reprogrammed in 2020. Patient-derived fibroblasts carrying the InDel variant were reprogrammed using a retroviral-based method with pMXs vectors encoding the four Yamanaka factors OCT3/4 (#17217, Addgene), SOX2 (#17218, Addgene), KLF4 (#17219, Addgene) and c-MYC (#17220, Addgene). All vectors were gifts from Shinya Yamanaka. Details on the viral reprogramming process are described in Haag et al.^21^ Fibroblasts derived from the patient carrying the p.G226R variant were reprogrammed using the non-integrative ReproRNA™-OKSGM Kit (5930, Stemcell Technologies), following the manufacturer's instructions. Emerging iPSC colonies were manually selected and cultured on Matrigel-coated (354277, Corning) plates in mTeSR medium (85850, Stemcell Technologies). Two iPSC clones were generated per patient. For the G226R variant, both clones were used in downstream experiments, while for the InDel variant, one clone per patient was selected for further analyses. Following reprogramming, all generated iPSC clones underwent karyotyping at the Institute of Medical Genetics and Applied Genomics in Tübingen to confirm the absence of major chromosomal abnormalities. The presence of the respective pathogenic variants was confirmed by PCR and subsequent Sanger sequencing (LGC Genomics) using variant-specific primers (G226R forward: 5′-TTGTAGCTCTCTCCCTCGTCA-3′, G226R reverse: 5′-AGATCGACACACGGACAGAT-3′; InDel forward: 5′-TGCTCATGGACTCAGCCTTC-3′, InDel reverse: 5′-GTGAGATGTCTGGGTGGGAAC-3′). For this purpose, genomic DNA was extracted using the QuickExtract™ DNA Extraction Solution (QE0905T, LGC Biosearch Technologies). To ensure mycoplasma-free cultures, routine screening was performed using the TaKaRa PCR Mycoplasma Detection Set (6601, TaKaRa Bio) according to the manufacturer's instructions. Finally, pluripotency of the iPSC clones was confirmed by RT-qPCR using the SensiFAST SYBR Lo-Rox One-Step Kit (74001, Bioline) and the ISOLATE II RNA Mini Kit (52072, Bioline) for RNA extraction, as well as by immunocytochemistry employing established pluripotency markers. Details on antibodies and their respective dilutions are provided in the Immunocytochemistry methods section. The RT-qPCR primers used for viral and RNA-based reprogramming are detailed in Haag et al.^21^ and Murti et al.,^22^ respectively.

### Site-specific integration of *NGN2* into the AAVS1 locus

Stable iPSC lines with inducible Neurogenin-2 (*NGN2*) expression were generated using a CRISPR/Cas9-mediated approach targeting the *AAVS1* safe-harbour locus. The pXAT2 plasmid (#80494, Addgene) encoded the Cas9 enzyme and an AAVS1-specific single guide RNA, while the pUCM-AAVS1-TO-hNGN2 plasmid (#105840, Addgene) carried a doxycycline-inducible *NGN2* transgene, the reverse tetracycline transactivator (rtTA), and a puromycin resistance cassette, all flanked by *AAVS1* homology arms. Both plasmids were kindly provided by Knut Woltjen and Michael Ward, respectively. iPSCs were nucleofected with 1 μg of each plasmid using the Nucleofector™ Transfection 2 b Device (Lonza) and the Human Stem Cell Nucleofector™ Kit 2 (Lonza), following the manufacturer's instructions. Cells were then plated onto Matrigel-coated wells in mTeSR medium supplemented with 10 μM Y-27632 (1254, Tocris) to promote survival. Puromycin (ant-pr-1, InvivoGen) selection (0.5 μg/mL for 72 h) was initiated after colony emergence to enrich for stably integrated clones. Positively selected lines were expanded, cryopreserved, and used for downstream applications.

### *NGN2*-based network culture differentiation

iPSCs were maintained in mTeSR medium with daily medium changes until neuronal induction. On the induction day, cells were pre-treated with 2.5 μg/mL doxycycline (D9891, Sigma–Aldrich) for 5 h to initiate *NGN2* expression, and 10 μM Y-27632 was added 1 h prior to dissociation to enhance cell survival.^23^^,^^24^ Following enzymatic detachment with accutase (A6964, Sigma–Aldrich), the cells were resuspended in neural stem cell medium (NSCM; 47.5% Neurobasal Medium (21103049, Gibco), 47.5% DMEM/F12 (21331-020, Gibco), 1% N2 supplement (N2-K, Capricorn), 2% NSC supplement (C21–H, Capricorn), 1% GlutaMAX (35050087, Gibco), 1% non-essential amino acids (P08-32100, PAN-Biotech GmbH), and 1% Penicillin/Streptomycin (15140122, Gibco) supplemented with 10 μM Y-27632, 3 μM DAPT (2634, Tocris), and 1 μg/mL doxycycline. The cells were then seeded at a density of 60 × 10^3^ per coverslip and 40 × 10^3^ per well of a 24-well MEA plate (24W700/100F-288, Multi Channel Systems). The following day, the medium was replaced with NSCM containing 10 μM DAPT, 10 μM PD0325901 (4192, Tocris), and 1 μg/mL doxycycline. From day 5 onward, differentiation was continued in NSCM without additional supplements, with medium changes twice per week. To eliminate proliferative precursor cells or incompletely converted iPSCs, the cells were additionally treated with 2 μM cytosine-β-D-arabinofuranoside (Ara-C; C1768, Sigma–Aldrich) from day 8 to day 11. For all electrophysiological experiments, *NGN2*-induced neurons (iNs) were co-cultured with primary mouse astrocytes, which were plated three days prior to induction at a density of 40 × 10^3^ cells per well. For immunostaining experiments of iN networks, iNs were cultured on poly-L-ornithine- (P3655, Sigma–Aldrich) and laminin-coated (L2020, Sigma–Aldrich) coverslips.

### *NGN2*-induced autaptic neuron generation

Autaptic cultures were generated by seeding iPSC-derived neurons at low density onto astrocyte-covered microislands. Microislands were prepared following established protocols by applying discrete dots of a coating solution onto agarose-coated 18 mm coverslips using a custom-built stamping tool.^25^, ^26^, ^27^ The coating solution consisted of 0.5 mg/mL poly-D-lysine (P6407, Sigma–Aldrich), 2 mg/mL collagen (C5533, Sigma–Aldrich), and 17 mM acetic acid (6755.1, Carl Roth GmbH + Co. KG) (1:1:3 ratio). Primary mouse astrocytes were plated at a density of 20 × 10^3^ cells/well in DMEM (41965039, Gibco) + 10% FBS (P30-3306, PAN-Biotech GmbH). Upon confluency, astrocyte microisland cultures were treated with 2 μM Ara-C for at least 24 h. For induction, iPSCs were treated with 10 μM Y-27632 for 1 h, washed with PBS (14190094, Gibco), and detached using accutase. After centrifugation, the cell pellet was resuspended in NSCM supplemented with 10 μM Y-27632 and 2 μg/mL doxycycline and plated on Matrigel-coated 6-well plates at 300 × 10^3^ cells/well. The medium was refreshed over the following two days with NSCM + doxycycline (2 μg/mL). On day 4, 2 μM Ara-C was added for 24 h. On day 5, iNs were harvested and cryopreserved in knockout serum replacement (10828028, Gibco) containing 10% DMSO (A3672, PanReac AppliChem). Freshly thawed iNs were plated onto confluent astrocyte microislands at a density of 5–7 × 10^3^ cells/well in NSCM supplemented with 10 ng/mL BDNF (450-02, PeproTech), CNTF (450-02, PeproTech), GDNF (450-10, PeproTech), and 0.5% FBS. Medium was changed weekly.

### Immunocytochemistry

For immunocytochemical stainings, cells were washed with PBS and fixed in 4% paraformaldehyde (PFA) for 10 min at room temperature (RT). After fixation, cells were again washed with PBS and stored at 4 °C. To block non-specific binding, coverslips were incubated for 1 h at RT in blocking solution containing 0.2% Triton X-100 (9036-19-5, Sigma–Aldrich) and 10% normal goat serum (NGS; 566380, Sigma–Aldrich) in PBS. Cells were then washed and incubated with primary antibodies overnight at 4 °C. The next day, secondary antibodies were applied for 1 h at RT in the dark. Coverslips were mounted using DAPI Fluoromount-G (0100-20, Southern Biotech). All antibodies were diluted in a 1:10 solution of blocking buffer in PBS.

Pluripotency was confirmed using rabbit anti-OCT4 (1:2000; ab19857, Abcam, RRID:AB_445175), rabbit anti-SOX2 (1:2000; ab97959, Abcam, RRID:AB_2341193), mouse anti-SSEA4 (1:2000; ab16287, Abcam, RRID:AB_778073), and mouse anti-TRA-1-60 (1:2000; ab16288, Abcam, RRID:AB_778563) primary antibodies.

Synapse quantification was performed using rabbit anti-Synapsin (1:1000; ab1543, Sigma–Aldrich, RRID:AB_2200400) in combination with chicken anti-MAP2 (1:2000; ab5392, Abcam, RRID:AB_2138153).

For Bassoon–Homer colocalisation stainings, the protocol was modified as follows: Tris-buffered saline (TBS; 150 mM NaCl, 50 mM Tris–HCl, pH 7.6) containing 0.1% Triton X-100 (TBS-Tx) was used instead of PBS throughout the procedure. Non-specific binding was blocked with 5% NGS in TBS-Tx. Primary antibodies were diluted in blocking solution and included rabbit anti-Homer1 (1:500; Synaptic Systems, 160003, RRID:AB_887730) and mouse anti-Bassoon (1:500; Enzo Life Sciences, ADI-VAM-PS003-F, RRID:AB_1659573), together with chicken anti-MAP2 (1:2000; ab5392, Abcam, RRID:AB_2138153).

All secondary antibodies were used at a 1:1000 dilution (A-11001, RRID:AB_2534069; A-11011, RRID:AB_143157; A-11039, RRID:AB_2534096; A-11008, RRID:AB_143165; A32731TR, RRID: AB_2866491; A-11004, RRID: AB_2534072; A-21449, RRID: AB_2535866, Thermo Fisher).

Images of pluripotency markers were acquired on a Zeiss Axio Imager Apotome 2 fluorescence microscope (Carl Zeiss AG). Synapse quantification in autaptic and network cultures was performed using images acquired with a Leica TCS SP8 confocal microscope (Leica Microsystems) using tile scan and z-stack modes. Z-stacks were converted to maximum intensity projections in ImageJ 1.54 (National Institutes of Health) before analysis. An automated analysis pipeline was created with CellProfiler 4.2.6. (Broad Institute). Soma and neurite regions were defined based on MAP2 signal using adaptive thresholding. Synapses were identified by thresholding the synapsin signal and applying a size filter to detect puncta. The same analysis pipeline was used for the detection of functional synapses based on Bassoon/Homer colocalisation. A MAP2-based neuronal mask ensured synapse quantification was limited to neuronal areas. In addition, neurite length was quantified to calculate synaptic density, expressed as synapsin or Bassoon/Homer puncta per 100 μm of neurite length.

### Western blot

For protein identification, 5 μg of total cell lysate per lane was loaded onto an 8% SDS–polyacrylamide gel. Following electrophoretic separation and transfer, Protran® Nitrocellulose Membranes were blocked with 5% skim milk powder and subsequently incubated overnight at 4 °C with the following primary antibodies: rabbit anti-*STX1B* (1:3000, 110403, Synaptic Systems, RRID:AB_887900), chicken anti-MAP2 (1:10,000, ab5392, Abcam, RRID:AB_2138153), or mouse anti-Vinculin (1:2000, V9131, Sigma–Aldrich, RRID:AB_477629), all diluted in 5% skim milk in PBS containing 0.1% Tween-20. After washing, membranes were incubated for 1 h at room temperature with HRP-conjugated secondary antibodies: goat anti-rabbit (Bio-Rad 1706515, RRID:AB_3752686), goat anti-chicken (Promega G135A, RRID:AB_430845), or goat anti-mouse (Bio-Rad 172-1011, RRID:AB_11125936), each diluted 1:10,000. Blots were developed using the Mini-PROTEAN® Tetra Cell according to the manufacturer's instructions. Signal quantification was performed using ImageJ. Syntaxin-1B expression levels were normalised to corresponding MAP2 signals in total lysates, and data were pooled from independent experiments.

### Whole-cell patch-clamp recordings of iPSC-derived neurons

Whole-cell patch-clamp recordings were performed at RT using an Axopatch 200 B amplifier (Molecular Devices), with Clampex 10.2 software (Molecular Devices) controlling data acquisition. Recording pipettes were pulled from borosilicate glass and had resistances between 2.5 and 4 MΩ. The intracellular solution contained 140 mM K-gluconate, 1 mM CaCl_2_, 10 mM EGTA, 2 mM MgCl_2_, 4 mM Na_2_ATP, and 10 mM HEPES (pH = 7.2, 305 mOsm), while the extracellular artificial cerebrospinal fluid consisted of 140 mM NaCl, 4.2 mM KCl, 1.1 mM CaCl_2_.2H_2_O, 1 mM MgSO_4_.7H_2_O, 0.5 mM Na_2_HPO_4_, 0.45 mM NaH_2_PO_4_, 5 mM HEPES, and 10 mM D-glucose (pH = 7.4, 300 mOsm). Series resistance was compensated by 80%, and only cells with access resistances below 12 MΩ were included in the analysis. Signals were sampled at 10 kHz and filtered using a 2 kHz low-pass Bessel filter. Data analysis was carried out using custom-written routines in MATLAB R2023b (MathWorks).^28^ The frequency and amplitude of miniature excitatory postsynaptic currents (mEPSCs) were quantified with the Mini Analysis Program V.6.0.7 (Synaptosoft Inc.). In autaptic cultures, iNs were held at a membrane potential of −70 mV, and synaptic responses were triggered by 2 ms depolarisation steps to 0 mV. Cells that failed to produce robust synaptic responses were included in the analysis of excitatory postsynaptic current (EPSC) peak amplitude and charge following single-pulse stimulation, but were excluded from protocols requiring sustained synaptic activity, such as high-frequency or paired-pulse stimulation. EPSC amplitude was determined by measuring the peak current, while synaptic charge was obtained by integrating the current over a 100 ms time window starting after the second capacitive artefact. Robust synaptic responses were defined by synaptic peak currents >160 pA and/or a synaptic charge >3 pC within a 100 ms time window.

Estimation of the readily releasable pool (RRP) was performed by forward extrapolation using the EQ plot.^29^ The synaptic charge released per stimulus was plotted against the cumulative charge release. Under the assumption that the majority of the RRP is depleted within the first three to five stimuli, a linear fit was applied to the first four data points. The x-axis intercept of this line was taken as the RRP estimate. At this early time point, the influence of newly recruited vesicles is presumed to be negligible. The vesicular release probability (P_VR_) was calculated as the ratio of the charge of the first EPSC (Q_EPSC1_) to the estimated RRP charge (Q_RRP_): P_VR_ = Q_EPSC1_/Q_RRP_. To determine the vesicle recruitment rate, synaptic responses were elicited with 100 stimuli at 20 Hz to deplete the RRP and the SMN fit by Schneggenburger et al. was applied to assess vesicle replenishment.^30^ For this, cumulative charge release was plotted against stimulus number. Towards the end of the train (last 20 stimuli), the response enters a linear steady-state phase in which vesicle recruitment balances ongoing release. The slope of this linear phase served as a measure of the vesicle recruitment rate.

For recordings in conventional (non-autaptic) cultures, mEPSCs were measured at a holding potential of −80 mV, which corresponds to the Cl^−^ equilibrium potential. Tetrodotoxin (TTX, 1 μM) was added to block action potentials (AP) and isolate mEPSCs. Passive and active membrane properties were recorded in current-clamp mode. The measured liquid junction potential of +15 mV was corrected, resulting in an effective holding potential of −70 mV. APs were evoked by depolarising current injections from −5 to +135 pA. The first spike at the +65 pA step, which consistently elicited APs, was used for analysis of active membrane properties including AP amplitude, threshold, half-width and after hyperpolarisation.

### Whole-cell patch-clamp recordings of mouse hippocampal neuronal cultures

Mouse hippocampal neuronal cultures were prepared as previously described.^31^ Briefly, the hippocampi were isolated from E18 embryos and digested by 2.5% trypsin (Invitrogen). Dissociated neurons were plated on 13 mm coverslips at a density of 1.2 × 10^5^ cells/coverslip. The lentiviral constructs that were used to transduce the hippocampal cultures at DIV 3–4 were created from a customised construct (pSyn1-NLS-GFP-P2A-hSTX1B), kindly provided by Prof. Christian Rosenmund.^20^ Whole-cell patch-clamp recordings were performed in transduced hippocampal cultures at DIV 13–19. The recording pipettes were filled with ‘intracellular solution’ containing 136 mM KCl, 4.6 mM MgCl2, 17.8 mM HEPES, 1 mM EGTA, 4 mM Na_2_ATP, 0.3 mM Na2GTP, 12 mM creatine phosphate and 50 U/ml phosphocreatine kinase (pH 7.4, 300 mOsm), and had a resistance of 3–5 MΩ. The extracellular solution contained 140 mM NaCl, 2.4 mM KCl, 10 mM HEPES, 10 mM glucose, 2 mM CaCl_2_, and 4 mM MgCl_2_ (pH 7.4, 300 mOsm). TTX (1 μM) and picrotoxin (5 μM) were added into the extracellular solution for recording of mEPSCs. Cells were held at −70 mV. Signals were sampled at 20 kHz and low-pass filtered at 1 kHz. Data analysis was performed using Clampfit 10.7.

### Multiwell-MEA recordings of iN networks

Neuronal network activity was recorded using a 24-well Multiwell-MEA system (Multi Channel Systems), with each well equipped with 12 gold electrodes (100 μm diameter, 700 μm spacing). Signals were acquired at a sampling rate of 20 kHz. Raw traces were processed using a fourth-order low-pass Butterworth filter (cutoff frequency: 3500 Hz) and a second-order high-pass Butterworth filter (cutoff frequency: 100 Hz). The system maintained a constant temperature of 37 °C throughout the recordings. Prior to each recording session, a 10-min equilibration period of the plate ensured thermal stability, followed by a 5-min data acquisition period. To verify network culture quality across batches, iNs were seeded in parallel onto glass coverslips and regularly inspected via microscopy. Cultures displaying atypical morphology (dense clustering or failed neuronal induction) were excluded from the dataset. Spike and burst detection relied on the built-in algorithm of the Multiwell-Analyzer software V.2.0.6.0 (Multi Channel Systems). To determine baseline noise, the standard deviation of the raw signal was calculated from 50 segments of 100 ms each, and spike thresholds were set at ± 5 times this value. Bursts were defined by the following criteria: a minimum of 5 spikes, inter-spike intervals below 10 ms for burst initiation and termination, a minimum burst duration of 50 ms, and a minimum inter-burst interval of 50 ms. Network bursts were identified when at least 6 electrodes exhibited overlapping burst activity. Wells not meeting the minimum activity thresholds (burst rate ≥0.4 bursts/min and mean firing rate ≥0.1 Hz) were excluded from downstream analysis. Further data analysis was conducted using a custom-written Python pipeline.^32^ For each well, spike and burst counts were averaged across all electrodes. To reduce data dimensionality and emphasise key sources of variation, a principal component analysis (PCA) was performed using the scikit-learn Python package from Pedregosa et al.^33^ Input features were z-scored to normalise variance across parameters. PCA was applied to seven features: total spike count, interburst spike ratio, NB count per minute, NB duration, NB inter-burst interval, NB spike frequency, and the coefficient of variation of the NB inter-burst interval. The first two principal components were ultimately used for visualisation.

### Bulk RNA sequencing

For bulk RNA sequencing, iN cultures were generated as network cultures. The mouse astrocyte feeder layer was prepared by plating 300 × 10^3^ astrocytes per well in a 6-well plate three days prior to iPSC seeding. iPSCs were then plated onto the astrocyte feeder layer at 250 × 10^3^ cells per well and differentiated as previously described for the method of network culture differentiation. RNA was extracted from three independent batches of differentiation at DIV 28 using the ISOLATE II RNA Mini Kit according to the manufacturer's instructions. 1 μg of RNA per sample, derived from a heterogeneous mixture of human and mouse material, was subjected to poly-A enrichment for mRNA, sequenced by the Institute for Medical Genetics and Applied Genomics (IMGAG) and analysed by the Quantitative Biology Center (QBiC), two partner institutes of the NGS Competence Center Tübingen (NCCT). To optimise the analysis of the data, filtering steps were performed prior to read mapping and downstream analysis. Human reads were filtered from the original FASTQ files using the nf-core/detaxizer pipeline V.1.1 (dev = v1.1.0dev-g2fac8db, https://nf-co.re/detaxizer).^34^ In brief, taxonomic classification of all input reads was performed using Kraken2 V.2.1.3^35^ with the nt database from November 29, 2023. The analysis revealed that, at the domain level, on average, 87% of reads were assigned to Eukaryota, with 56% on average classified as *Mus musculus* and 4% on average as *Homo sapiens*, reflecting the co-culture system. These reads classified as *H. sapiens* were then extracted as FASTQ files for downstream processing with nf-core/rnaseq. Approximately 10% of reads could not be confidently assigned at the domain or kingdom level, likely due to low-complexity or non-specific k-mer content. Reads assigned to Bacteria, Archaea, Viruses, or artificial sequences were negligible (<0.04%) and an additional ∼0.2% remained unclassified due to the absence of matching k-mers. The obtained files, used for further analysis, contained 0.4–5.4 million reads from which 89–97% were assigned to *H. sapiens*. Quality control, read mapping, and counting were performed using the nf-core/rnaseq pipeline V.3.12.0 (https://nf-co.re/rnaseq).^36^ Quality control assessment of the raw data was performed using FastQC V.0.11.9^37^ and RSeQC V.3.0.1.^38^ Reads were mapped to the reference genome (GRCh38) using the STAR aligner V.2.7.10a.^39^ Quantification of raw gene expression was performed with Salmon V.1.10.1.^40^ Subsequently, differential gene expression, gene ontology (GO), and pathway analysis were performed with the Nextflow-based rnadeseq pipeline V.2.4 (https://github.com/qbic-pipelines/rnadeseq). This included the differential expression analysis using the DESeq2 R package V.1.40.2,^41^ by which statistics including adjusted p-values (padj) for each gene were calculated using the Benjamini-Hochberg method. The identification of differentially expressed genes (DEG) was determined by a padj <0.05 and subsequently filtered for biological relevance using a cut-off of ±1.5-fold change in gene expression. Finally, the gProfiler2 V.0.2.2 was used for subsequent pathway and GO enrichment analysis^42^ as part of the above mentioned rnadeseq pipeline. Terms with padj <0.05, calculated using the Benjamini-Hochberg FDR method, were considered enriched. The data discussed in this publication have been deposited in NCBI's Gene Expression Omnibus^43^ and are accessible through GEO Series accession number GSE329848 (https://www.ncbi.nlm.nih.gov/geo/query/acc.cgi?acc=GSE329848).

### Ethics

The study was approved by the Ethics Committee of the Medical Faculty at the University of Tübingen (approval number 428/2023BO2) and was conducted in accordance with the 2024 Declaration of Helsinki. Written informed consent was obtained from all donors, or from a parent or legal guardian where the donor was a minor, prior to skin biopsy. All animal procedures were carried out in accordance with institutional guidelines and German animal welfare legislation and were approved by the responsible local authorities (approval number N14-22 M). All experiments reported in this study were performed between 2020 and 2025.

### Statistical analyses

Statistical data analysis was carried out using GraphPad Prism V.5.04. To assess the distribution of the datasets, both the Shapiro–Wilk and Kolmogorov–Smirnov tests were applied. Results from these tests indicated that all datasets followed a non-parametric distribution. Therefore, the statistical significance of multiple groups was tested using the Kruskal–Wallis test (unpaired), followed by Dunn's test for multiple comparisons. For analyses involving more than one independent variable, a two-way or mixed-effect ANOVA with 10,000 permutations was employed to determine statistical significance, followed by a permutation-based independent t-test with Holm's multiple comparison correction. Outliers were determined based on the 1.5-fold interquartile range (IQR) method and were removed only following a careful determination that they stemmed from experimental errors rather than genuine biological variability. Statistical significance is represented as follows: ∗p < 0.05, ∗∗p < 0.01, and ∗∗∗p < 0.001. Sample size planning was done using Cohen's f = 0.4 as a pragmatic smallest effect size of interest (α = 0.05, β = 0.2), which indicated a sample size (N) of 22 per group; N denotes independent neuronal cultures for MEA and immunohistochemistry experiments, and individual recorded cells for patch-clamp experiments. This target was reached for the majority of datasets, but not for all; the exact N and n for every comparison are given in Supplementary Table S1. All iPSC lines were labelled with internal laboratory identifiers throughout culture, data acquisition and analysis, so that the experimenter remained blinded as to whether a given line was control- or patient-derived. Within each experimental paradigm, all cell lines were differentiated, recorded and analysed in parallel, within the same experimental period and under identical culture and recording conditions. Different assays were performed at different times, but no individual cell line was examined in a time window separate from the others.

### Role of funders

The funders had no role in study design, data collection, data analysis, interpretation of data, or writing of the report.

## Results

### Generation and neuronal differentiation of iPSC lines from patients with *STX1B* variants

**Fig. 1:**
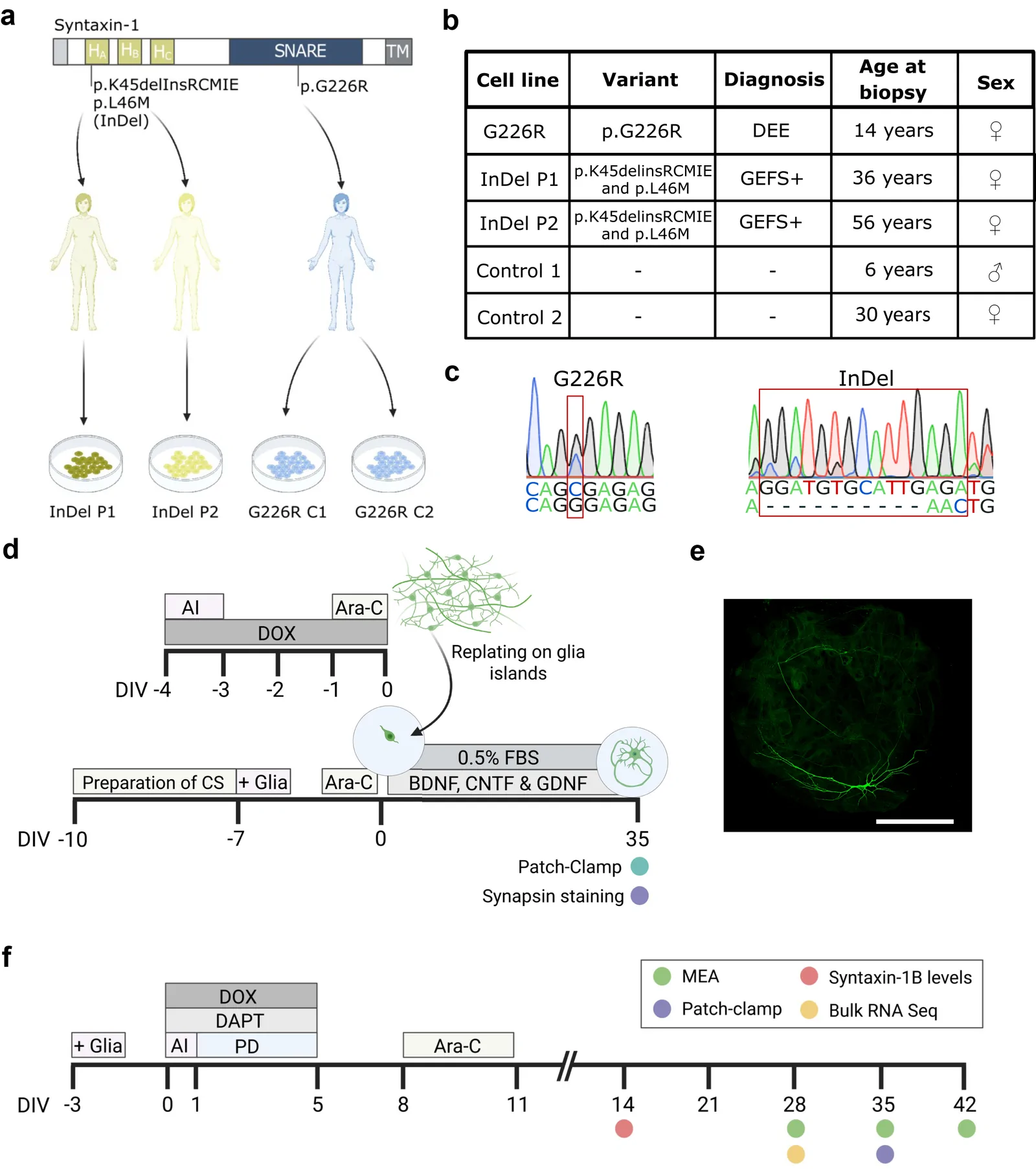
**Overview of the study design and iPSC-differentiation protocols**. **a** Schematic representation of the human syntaxin-1B protein structure indicating the location of individual patient-specific variants. The corresponding iPSC lines generated from each patient carrying the respective variant are shown below. Created with Biorender.com. **b** Table summarising characteristics of each iPSC line, including the specific *STX1B* variant, as well as the clinical diagnosis, age at skin biopsy, and sex of the respective donor. **c** Representative Sanger sequencing results of both *STX1B* variants, verifying the presence of the variant sites in the corresponding iPSC lines. **d** Timeline of iPSC differentiation into autaptic cultures. iPSCs were initially pre-differentiated into a neuronal network via doxycycline-inducible *NGN2* overexpression. In parallel, microglial islands were prepared. Once the microglial islands reached confluency, the neuronal network was disrupted, and single neurons were replated onto the microglial islands. Downstream analyses, including patch-clamp and immunostainings, were performed at DIV 35 (post-replating). Created with Biorender.com. **e** MAP2-stained autaptic neuron forming a self-innervating network. Scale bar: 200 μm. **f** Schematic timeline of neuronal network differentiation. iPSC-derived neurons were plated directly onto astrocyte feeder layers to promote neuronal differentiation and synapse formation. The timing of downstream analyses varied depending on the experimental readout and is indicated by colour-coded markers along the timeline. Created with Biorender.com. Abbreviations: AI, apoptosis inhibitor (Y-27632); Ara-C, cytosine-β-D-arabinofuranoside; DOX, doxycycline; CS, coverslips; FBS, foetal bovine serum; BDNF, brain-derived neurotrophic factor; CNTF, ciliary neurotrophic factor; GDNF, glial cell line-derived neurotrophic factor; Dapt, N-[N-(3,5-Difluorophenacetyl)-L-alanyl]-S-phenylglycine t-butyl ester; PD, PD0325901.

We generated iPSCs from three different patients, each carrying a heterozygous pathogenic variant in the *STX1B* gene (Fig. 1a). Two related patients (mother and daughter) carry the same complex InDel variant (c.133_134insGGATGTGCATTG/p.K45delinsRCMIE and c.135_136AC > GA/p.L46M), which is located in the first helix of the regulatory H_abc_-domain. The third patient carries a *de novo* missense variant (c.676G > C/p.G226R) in the SNARE motif. For the latter, we pooled iPSCs from two clones, while using one clone from each individual carrying the InDel variant. The control group consisted of iPSC lines derived from two independent healthy individuals of both sexes. Fig. 1b summarises the clinical background of the individuals included in the study. While the donor carrying the G226R missense variant presents with a severe epilepsy phenotype of DEE, the two patients with the InDel variant have been diagnosed with the milder syndrome GEFS+ and have become seizure-free without further medical treatment.

Following the reprogramming of fibroblasts into patient-specific iPSCs, all cell lines were screened for a normal karyotype and the expression of pluripotency markers (Supplementary Fig. S1). In addition, Sanger sequencing confirmed the presence of the respective pathogenic variants in patient cell lines (Fig. 1c). To study synapse physiology in depth, we employed the autaptic culture system, in which neurons are grown in isolation on astrocyte islands, allowing for high experimental control during patch-clamp recordings to assess various synaptic properties. Alternatively, iPSCs were also differentiated into neuronal network cultures. In both approaches, we leveraged the *NGN2*-overexpression for differentiation, which yields predominantly cortical-like excitatory neurons. The differentiation protocols for the generation of iNs for autaptic and network cultures and the associated experimental time points are shown in Fig. 1d and f, respectively. Synaptic parameters were evaluated by whole-cell patch-clamp recordings at DIV 35, while transcriptomic profiling by bulk RNA sequencing was performed at DIV 28. MEA recordings were initiated at DIV 28 and continued until DIV 42, during which network activity was robust and remained relatively stable (Supplementary Fig. S4d). The absence of major changes in network dynamics within this period suggests that neuronal and synaptic properties are largely maintained, supporting the comparability of data collected at these different, yet closely related, time points.

For simplicity, G226R- and InDel-containing iNs are hereafter referred to as G226R-iNs and InDel-iNs, respectively.

### *STX1B* InDel is associated with reduced Syntaxin-1B protein levels

The integrity of a protein's structure is critically dependent on its amino acid sequence, with changes potentially leading to misfolding, destabilisation, or degradation. In particular, the large *STX1B* InDel variant, involving the alteration of six consecutive amino acids, has the potential to significantly impair the structural stability of syntaxin-1B.

**Fig. 2:**
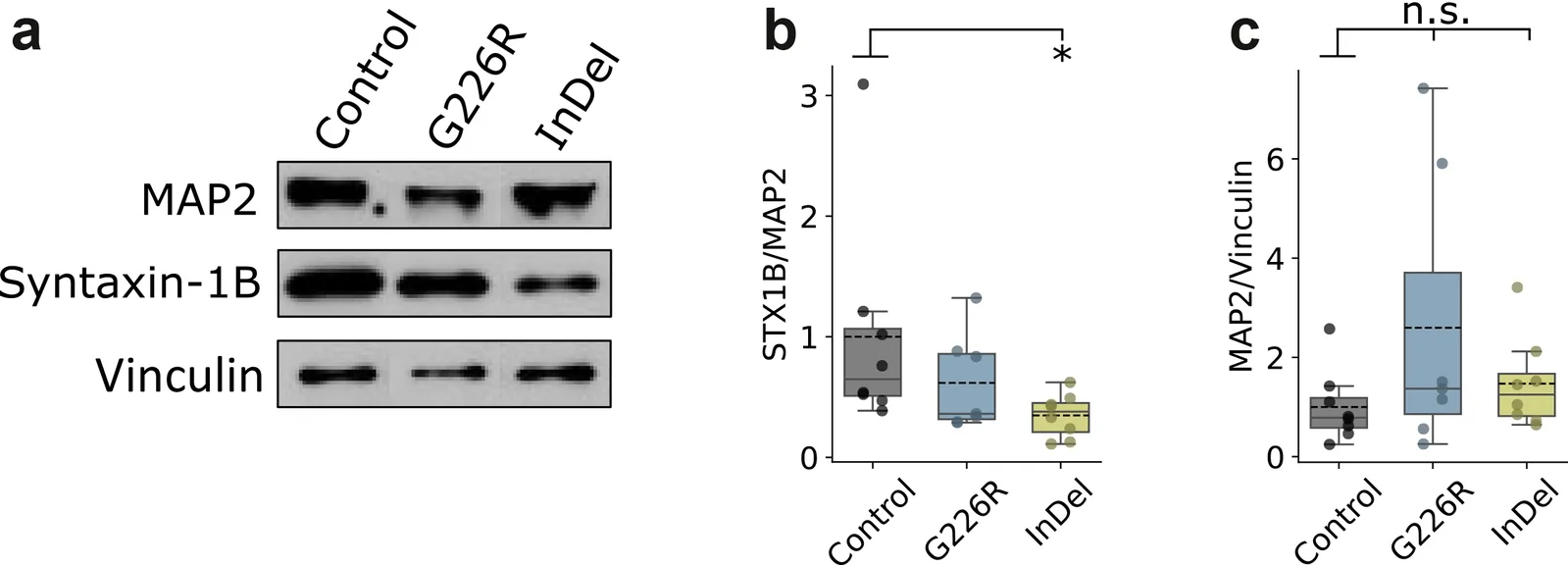
**Syntaxin-1B protein levels in iN networks**. **a** Representative Western blot showing syntaxin-1B protein levels in iN networks. Vinculin served as loading control. **b** Quantification of syntaxin-1B protein levels normalised to MAP2. The resulting syntaxin-1B/MAP2 ratios were further normalised to control and are presented as relative protein levels. InDel-iNs exhibit a significant reduction in syntaxin-1B levels compared to control, whereas G226R-iNs show no significant difference. **c** Quantification of MAP2 protein levels normalised to Vinculin to assess neuronal content and maturation. No significant differences were observed between conditions. Data are visualised as boxplots, with whiskers indicating the range within 1.5 × the interquartile range. Solid lines mark the median, and dashed lines indicate the mean. Each dot represents one cell lysate. Data are based on four independent differentiations. n = 8 (control), 7 (G226R) and 8 (InDel), corresponding to the number of individual cell lysates. Statistical comparisons were performed using the Kruskal–Wallis test followed by Dunn's post hoc analysis; ∗p < 0.05.

To test this directly, we assessed syntaxin-1B protein levels in our human iNs by Western blot analysis (Fig. 2a). Syntaxin-1B band intensities were normalised to MAP2, and the resulting syntaxin-1B/MAP2 ratios are presented relative to the control group.

Consistent with our hypothesis, InDel-iNs exhibited a significant reduction in syntaxin-1B protein levels compared to controls (p = 0.022). In contrast, G226R-iNs did not show a significant difference relative to control (p = 0.40), indicating that this single amino acid substitution does not affect syntaxin-1B abundance under these conditions (Fig. 2b).

To further assess whether variability in neuronal differentiation or maturity could account for these differences, MAP2 levels were additionally normalised to the loading control protein Vinculin. While G226R samples displayed considerable variability, no significant differences in MAP2/Vinculin ratios were observed for neither InDel-iNs (p = 0.39) or G226R-iNs (p = 0.41) compared to controls (Fig. 2c). These results indicate that the reduced syntaxin-1B levels in InDel-iNs are unlikely to be explained by differences in neuronal content or maturation state, but instead are consistent with reduced protein stability, potentially arising from the structural consequences of the InDel variant, which affects a larger stretch of the protein sequence.

### Failure of high-frequency neurotransmission in *STX1B* G226R-iNs

**Fig. 3:**
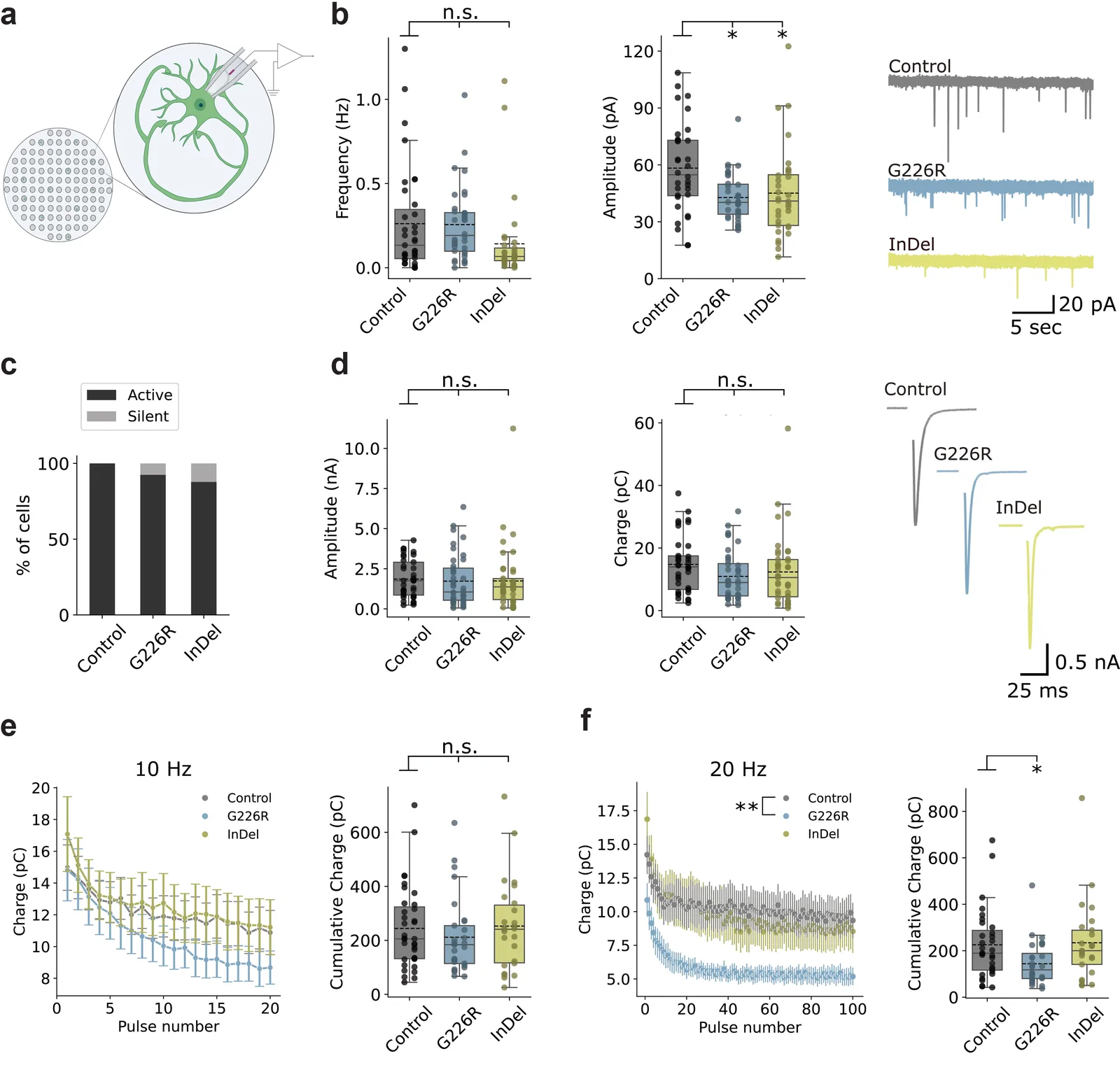
**Functional Consequences of *STX1B* Variants in Human Autaptic iNs**. **a** Schematic representation of a coverslip containing multiple microglia islands, each of which serves as a site for seeding individual iNs. A magnified view of one island illustrates an isolated neuron forming autaptic connections by establishing synapses onto itself. Patch-clamp recording of this neuron allows for high experimental control over both synaptic input and output. Created with Biorender.com. **b** mEPSC frequency and amplitude in autaptic iNs. The frequency of mEPSCs shows no significant difference between groups. The mEPSC amplitude of both patient variants is reduced compared to control. Representative traces of mEPSC recordings are shown on the right-hand side. **c** Proportion of silent synapses across groups. Silent synapses were defined as those with EPSC peak amplitudes below 160 pA and/or synaptic charge less than 3 pC within a 100 ms time window. **d** AP-driven EPSC responses to single stimulation show no differences in peak amplitude or charge (representing the magnitude of the synaptic response over time) between *STX1B* variants. Representative EPSC traces are displayed on the right-hand side. **e** Autaptic iNs were stimulated at 10 Hz for 20 pulses. The EPSC charge is plotted as a function of pulse number to depict the progressive rundown of synaptic release (left). No significant differences were observed between *STX1B* variants in the cumulative charge released during these pulses (right). **f** High-frequency stimulation was performed at 20 Hz for 100 pulses. The EPSC charge plotted against pulse number demonstrates synaptic release over time (left), while the cumulative charge released within the first 20 pulses is shown on the right. The G226R variant exhibits a significant reduction in cumulative charge release compared to controls. Data are visualised as boxplots, with whiskers indicating the range within 1.5 × the interquartile range. Solid lines mark the median, and dashed lines indicate the mean. Each dot represents a single cell. Data are based on four independent differentiations. n = 31–37 (control), 28–40 (G226R) and 25–41 (InDel). Statistical comparisons were performed using the Kruskal–Wallis test followed by Dunn's post hoc analysis; comparisons in **f** were done using permutation-based mixed-design ANOVA followed by post hoc permutation-based independent t-test with Holm's multiple comparison correction; ∗p < 0.05. Abbreviations: n.s., not significant.

To assess the functional impact of *STX1B* variants in autaptic iNs, whole-cell patch-clamp recordings were performed. The minimalistic design of this model system enables the recording of spontaneous synaptic vesicle release as well as the induction of action potential (AP)-driven synaptic responses, serving as a powerful tool to assess synaptic physiology at the single-cell level (Fig. 3a). Differentiation via *NGN2* overexpression yields predominantly glutamatergic neurons, allowing us to focus on excitatory synaptic responses. mEPSC analysis suggested subtle differences in frequency across groups, but these did not reach statistical significance (p = 0.53 and p = 0.058, for G226R and InDel, respectively) (Fig. 3b). In contrast, both patient-derived lines exhibited significantly reduced mEPSC amplitudes (p = 0.011 and p = 0.017, for G226R and InDel, respectively) (Fig. 3b). In most iNs we could elicit evoked synaptic responses, while a small percentage of patient-derived cells exhibited insufficient responses to a brief depolarising pulse (Fig. 3c). However, the peak amplitudes of these evoked EPSCs were similar across all groups, with no significant differences observed (p = 0.47 and p = 0.45, for G226R and InDel, respectively) (Fig. 3d). Likewise, the total charge transfer, reflecting both the magnitude and duration of the synaptic event, did not differ significantly (p = 0.09 and p = 0.17, for G226R and InDel, respectively) (Fig. 3d).

Short-term plasticity was evaluated using repetitive stimulation at 10 Hz (Fig. 3e) and 20 Hz (Fig. 3f ). At both frequencies, all groups displayed synaptic depression. Notably, G226R-iNs exhibited a significant reduction in charge transfer at 20 Hz (group effect F(2,81) = 3.55, permutation p = 0.028, main pulse effect F(98,7938) = 17.49, p = 0.0001, interaction F(196,7938) = 1.13, p = 0.11; post-hoc independent t-test G226R vs Control p = 0.011, InDel vs Control p = 0.79). Analysis of the cumulative charge released after 20 pulses at 20 Hz stimulation revealed an average charge release of 144.0 ± 18.0 pC in G226R-iNs (p = 0.038), compared to 225.5 ± 27.2 pC in controls (Fig. 3f, right panel). Normalisation of these repetitive synaptic responses suggested enhanced synaptic depression in patient-derived iNs at 20 Hz (group effect F(2,81) = 2.12, p = 0.13, main pulse effect F(98,10290) = 21.70, p = 0.0001, interaction F(294,7938) = 1.17, p = 0.024, Supplementary Fig. S2a). Consequently, only G226R displayed a significant deficit in maintaining high-frequency neurotransmission, as evidenced by the reduced charge transfer at 20 Hz stimulation.

This finding prompted us to further investigate factors influencing synaptic strength, such as the size of the RRP and P_VR_. Assuming a constant P_VR_, an altered size of the RRP would have direct consequences on the initial synaptic response. In line with this assumption, G226R-iNs exhibited a reduced charge release during the first synaptic response relative to controls (G226R: 10.9 ± 1.2 pC, Control: 14.7 ± 1.4 pC; p = 0.09) (Fig. 3d, right panel). However, as previously noted, this synaptic deficit only reached statistical significance under repetitive stimulation at 20 Hz (p = 0.038) (Fig. 3f ).

**Fig. 4:**
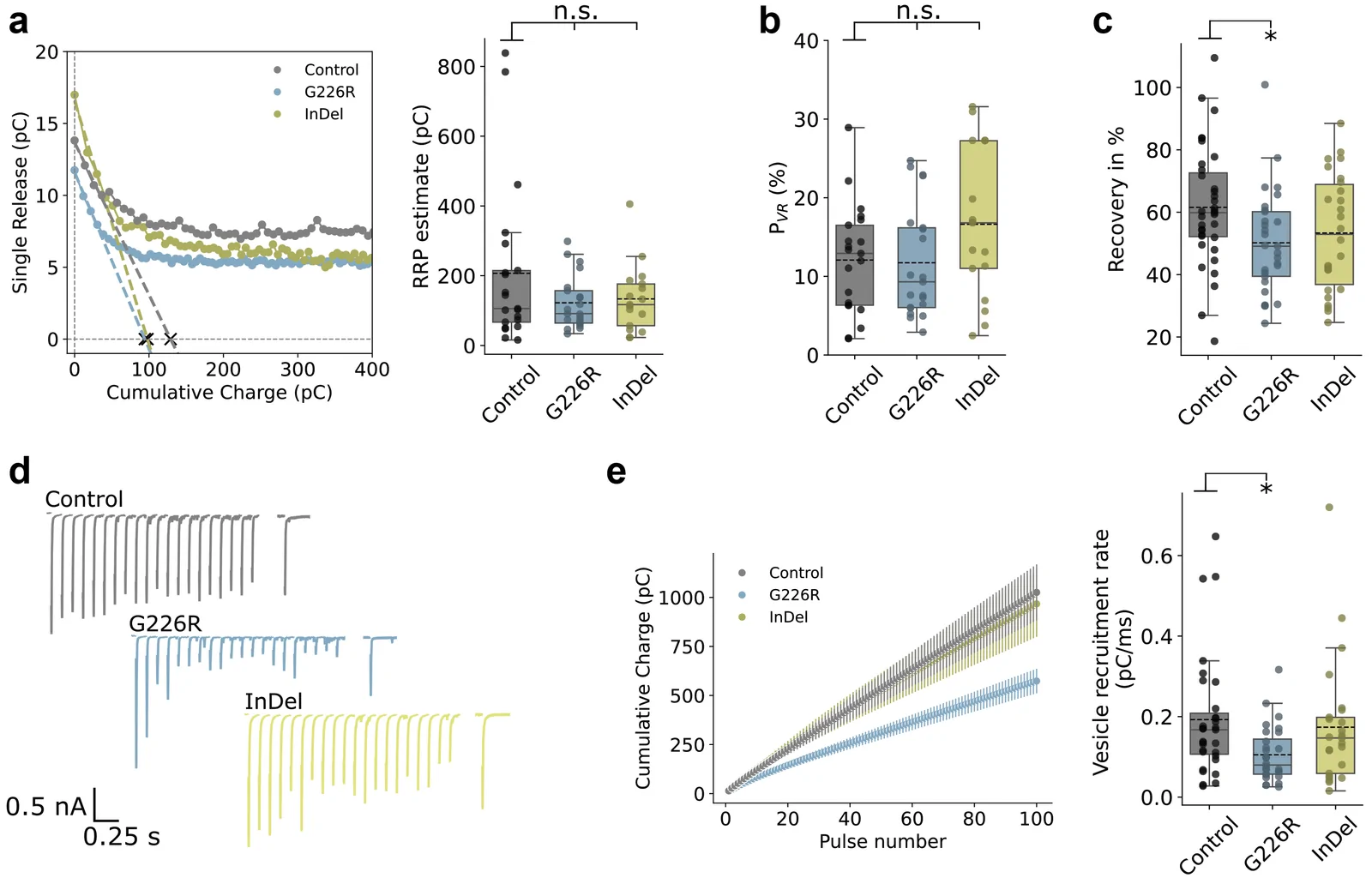
**The Impact of *STX1B* Variants on Synaptic Vesicle Dynamics**. **a** Estimation of the RRP was performed using the EQ method. This approach involves plotting individual responses from a stimulus train against the cumulative release. Assuming that most of the RRP is depleted within the first three to five pulses, a linear fit is applied to the first four data points, with the x-intercept indicating the RRP size estimate. The estimated RRP size did not differ significantly between groups. **b** The vesicular release probability of autaptic iNs shows no significant differences across the groups. **c** Recovery of EPSC amplitude measured 2 s after a 10 Hz stimulation train. The amplitude of the recovery response was normalised to the amplitude of the first synaptic event in the pulse train and showed a significantly reduced recovery compared to the control group. **d** Representative traces illustrate responses during the 10 Hz pulse train and the following recovery pulse. Note that the recovery pulse was delivered 2 s after the train stimulation. The corresponding 2 s interval is visually shortened for figure clarity. **e** Calculation of the vesicle recruitment rate was performed using the SMN fit. The cumulative charge released over 100 pulses at 20 Hz was plotted, and a linear regression was fitted to the last 20 data points. The slope of this fit represents the vesicle recruitment rate, which is significantly decreased in the G226R variant. Data are visualised as boxplots, with whiskers indicating the range within 1.5 × the interquartile range. Solid lines mark the median, and dashed lines indicate the mean. Each dot represents a single cell. Data are based on four independent differentiations. n = 21–35 (control), 21–29 (G226R) and 17–26 (InDel). Statistical comparisons were performed using the Kruskal–Wallis test followed by Dunn's post hoc analysis; ∗p < 0.05. Abbreviations: n.s., not significant; RRP, readily releasable pool.

To estimate the RRP size, we applied the EQ plot (Fig. 4a, left panel), which assumes rapid RRP depletion within the initial pulses of a high-frequency stimulus train, with a negligible contribution of newly recruited vesicles at this stage (see Methods). Using this method, we found no differences in RRP size between controls and *STX1B* variants (p = 0.91 and p > 0.99, for G226R and InDel, respectively) (Fig. 4a, right panel). Another commonly used method for estimating RRP size is the SMN fit, which assumes a constant rate of vesicle recruitment throughout the stimulus train. However, since this assumption was not consistently met in our recordings, we did not pursue this approach further. Finally, the P_VR_ as well as the paired-pulse ratio at varying inter-pulse intervals (IPI) did not differ across groups (P_VR_: p > 0.99 and p = 0.32; IPI50 ms: p = 0.67 and p = 0.45; IPI100 ms: p > 0.99 and p > 0.99; IPI200 ms: p = 0.85 and p = 0.17; IPI1000 ms: p = 0.69 and p > 0.99, for G226R and InDel, respectively) (Fig. 4b and Supplementary Fig. S2b). This suggests that neither the size of the RRP nor the P_VR_ is causative for the reduced charge release upon repetitive stimulation in G226R.

Instead, we considered impaired vesicle recruitment as a potential mechanism for the diminished synaptic responses. As the RRP is depleted during sustained activity, vesicles from the recycling pool are recruited to replenish the RRP. G226R-iNs exhibited significantly reduced recovery following a 10 Hz pulse train, indicating a deficit in vesicle replenishment (Fig. 4c and d).

Although the SMN fit was not applicable for estimating RRP size in our recordings, it can be used to calculate the vesicle recruitment rate during the steady-state phase of the high-frequency stimulus train, once the RRP was fully depleted. Using the slope of the fit as a measure of the vesicle recruitment rate, this analysis revealed a significantly lower vesicle recruitment in G226R-iNs (p = 0.020 and p > 0.99, for G226R and InDel, respectively) (Fig. 4e), thus further corroborating the hypothesis of an impaired vesicle replenishment in G226R.

### *STX1B* InDel iNs display increased synaptic density

**Fig. 5:**
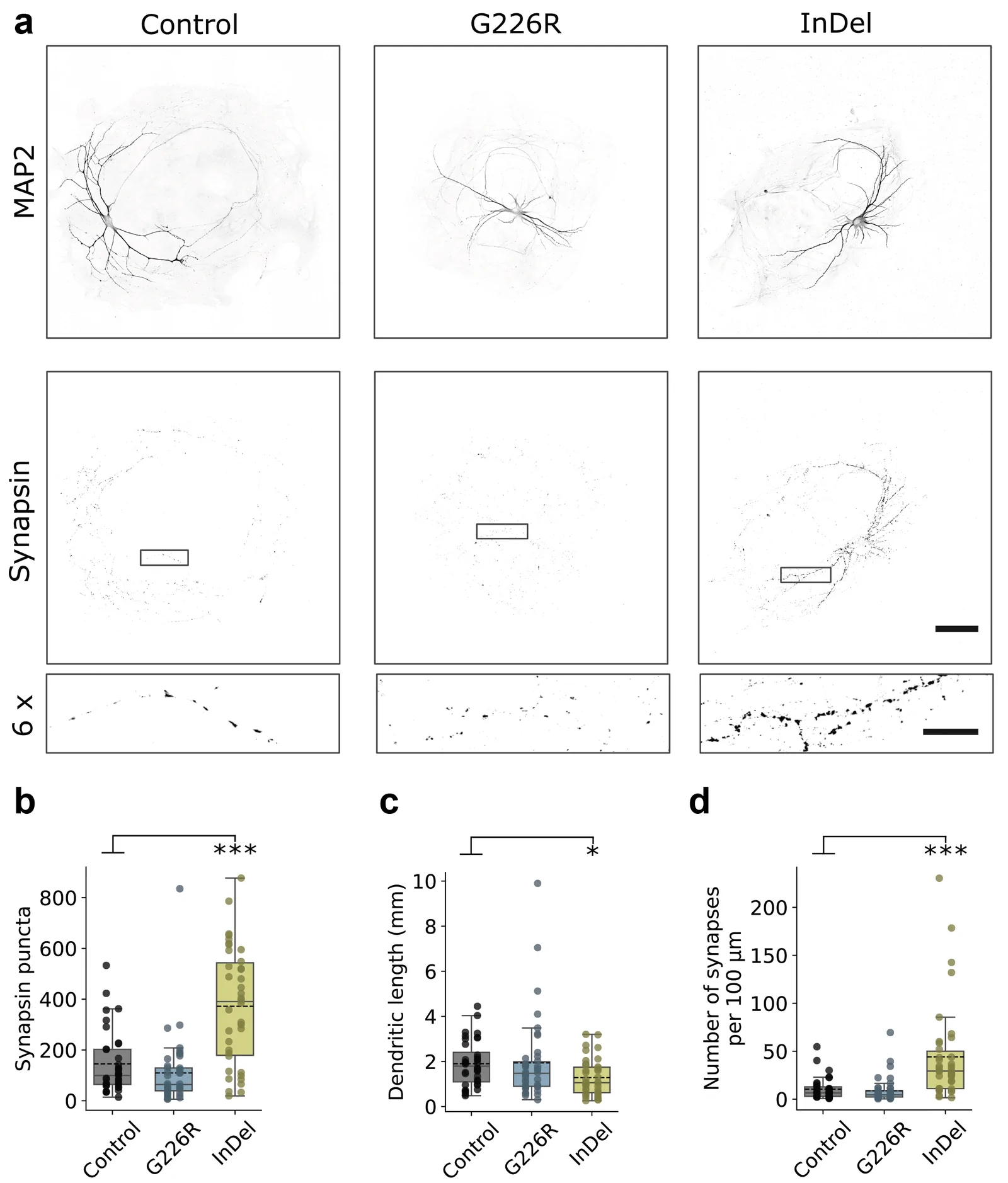
**Quantification of Synaptic and Dendritic Structures in Autaptic iNs**. **a** Representative immunofluorescence images showing dendrites and synapses labelled with MAP2 and synapsin, respectively (Scale bars: 100 μm for overview images; 20 μm for the synapsin magnification). **b** Quantification of synapses based on synapsin-positive puncta, showing a significantly increased number of synapses for InDel. **c** Measurement of dendritic length using a MAP2-based neuronal mask. InDel-derived neurons display a reduced dendrite length. **d** Synapse density quantified as the number of synapses per 100 μm dendrite length, with InDel-derived neurons exhibiting a significant increase. Data are visualised as boxplots, with whiskers indicating the range within 1.5 × the interquartile range. Solid lines mark the median, and dashed lines indicate the mean. Each dot represents a single cell. Data are based on four independent differentiations. n = 36 (control), 42 (G226R) and 38 (InDel). Statistical comparisons were performed using the Kruskal–Wallis test followed by Dunn's post hoc analysis; ∗p < 0.05, ∗∗∗p < 0.001.

Given that the *STX1B* InDel variant resulted in reduced syntaxin-1B protein levels but did not cause any detectable electrophysiological phenotype at the single-cell level, we next investigated whether this lack of phenotype could be attributed to compensatory adaptations, in particular to changes in synapse number. We therefore examined whether the *STX1B* variants might contribute to such morphological alterations that could potentially mask the electrophysiological phenotype. Autaptic iNs were immunostained for MAP2 and synapsin to label dendrites and presynaptic terminals, respectively (Fig. 5a). Quantitative analysis of synapsin puncta revealed a significantly increased number of synapses in the InDel variant compared to controls (p = 0.23 and p < 0.001, for G226R and InDel, respectively) (Fig. 5b), along with a reduction in neurite length (p = 0.55 and p = 0.011, for G226R and InDel, respectively) (Fig. 5c). This resulted in a highly significant increase in synaptic density for InDel-iNs, reaching 44.2 ± 8.1 synapses per 100 μm, in contrast to 10.5 ± 1.9 synapses per 100 μm in control neurons (p = 0.54 and p < 0.0001, for G226R and InDel, respectively) (Fig. 5d).

However, synapsin puncta alone do not necessarily represent functional synapses. To address this limitation, we performed co-immunostaining of the presynaptic marker bassoon and the postsynaptic marker homer. Representative images showed clear colocalisation of bassoon and homer puncta, consistent with the presence of bona fide synaptic contacts (Supplementary Fig. S3a). Quantification of pre- and postsynaptic colocalisation was next performed in iN network cultures (Supplementary Fig. S3b). This allowed us to assess whether the observed effect extends beyond autaptic single-cell systems to more complex neuronal networks, while taking advantage of a less time-intensive culture system. Consistent with our observations in autaptic neurons, we detected a significantly increased synaptic density in InDel cultures compared to controls, thereby validating the autaptic phenotype (p = 0.004 for Bassoon/Homer colocalisation per 100 μm) (Supplementary Fig. S3c). G226R-iNs did not exhibit any detectable changes in synapse number or density (p = 0.62 for Bassoon/Homer colocalisation per 100 μm).

### Both *STX1B* variants exhibit a network phenotype with varying severity

**Fig. 6:**
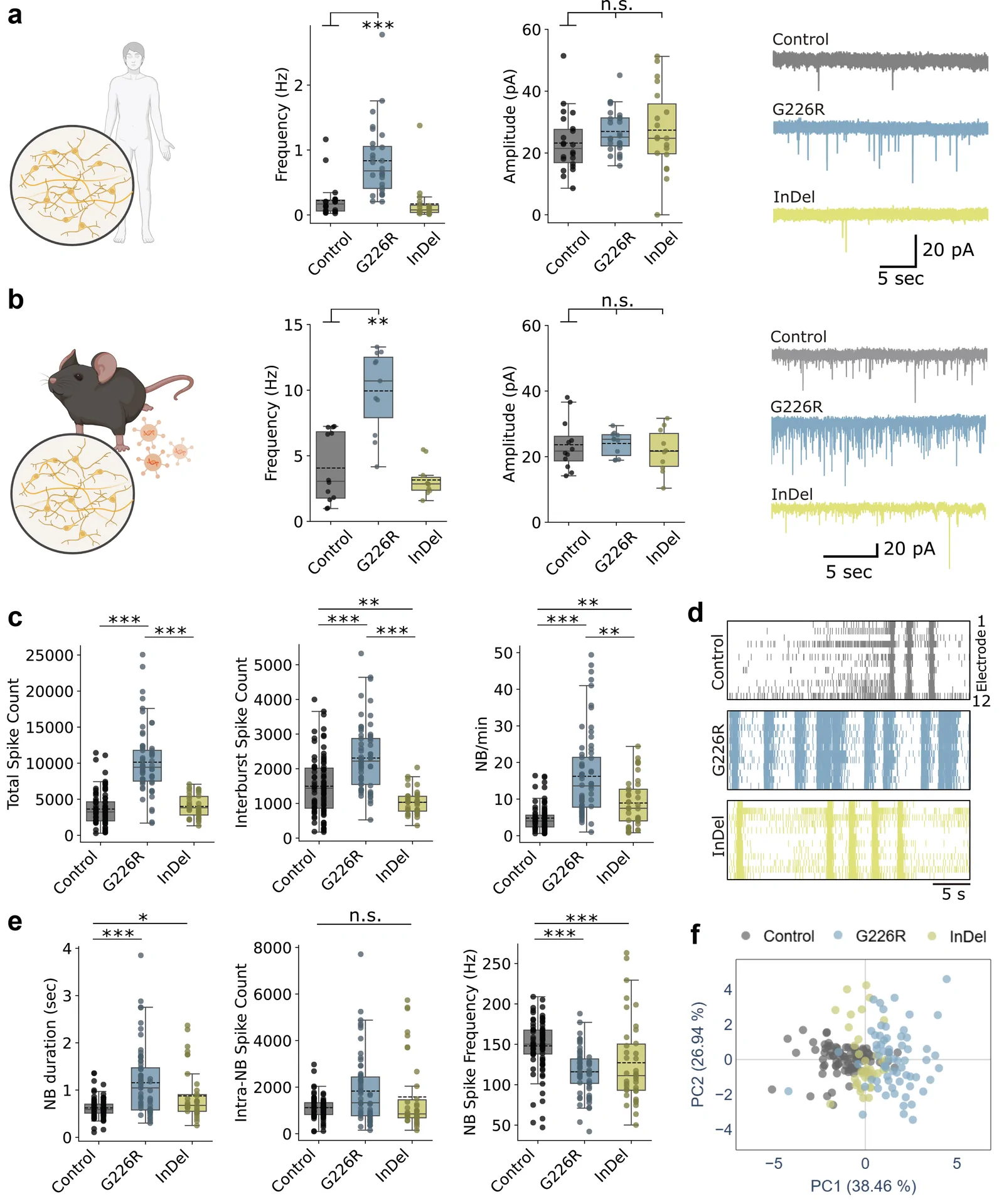
***STX1B* variants converge on a shared network phenotype**. **a** mEPSC frequency and amplitude recorded from human iN networks using whole-cell patch-clamp technique in the presence of 1 μM TTX. Representative traces are shown on the right-hand side. Data are based on three independent differentiations. n = 22 (control), 28 (G226R) and 19 (InDel). Parts of the figure were created with Biorender.com. **b** mEPSC frequency and amplitude in mouse hippocampal networks transduced with either WT *STX1B* or pathogenic variant constructs via lentiviral delivery. Whole-cell patch-clamp recordings were performed in the presence of 1 μM TTX. Example traces from each condition are shown on the right. n = 12 (control), 11 (G226R) and 10 (InDel). Parts of the figure were created with Biorender.com. **c** iN networks on multi-well MEAs at DIV 28. Patient-derived iNs showed significant alterations in several network parameters, including the total spike count, the interburst spike count (burst-unrelated spikes) and the number of network bursts (NB). Pathogenic variants led to an overall increase in network excitability. **d** Representative raster plots showing MEA activity over a 30 s interval, with each row representing a single electrode. **e** NB-specific parameters from multi-well MEA recordings at DIV 28. Both variants displayed elongated NB, but with unchanged intra-NB spike count, leading to a significant decrease in NB spike frequency. **f** PCA was performed using seven MEA parameters (total spike count, interburst spike ratio, NB count per minute, NB duration, NB inter-burst interval, NB spike frequency, and the coefficient of variation of the NB inter-burst interval) to identify group-specific clusters. Distinct clustering indicates differences in network activity profiles between groups. Data are visualised as boxplots, with whiskers indicating the range within 1.5 × the interquartile range. Solid lines mark the median, and dashed lines indicate the mean. For patch-clamp recordings of iN and mouse hippocampal networks (a and b), each data point represents an individual cell. iN-derived data were obtained from three independent differentiations. For MEA recordings (c and e), each dot corresponds to one well, with data collected from four to eight independent differentiations. n = 89 (control), 65 (G226R) and 38 (InDel). Statistical comparisons were performed using the Kruskal–Wallis test followed by Dunn's post hoc analysis; ∗p < 0.05, ∗∗p < 0.01, ∗∗∗p < 0.001. Abbreviations: n.s., not significant; NB, network burst; PC, principal component.

To determine whether the synaptic phenotypes differ between isolated neurons and connected networks, we additionally analysed mEPSCs in iN network co-cultures using whole-cell patch-clamp recordings in the presence of 1 μM TTX. While no significant difference in mEPSC frequency was detected in recordings of autaptic single neurons, neurons in iN networks carrying the G226R variant, but not the InDel, displayed a significantly increased mEPSC frequency compared to controls (p < 0.0001 and p = 0.56, for G226R and InDel, respectively) (Fig. 6a). The mEPSC amplitude, in contrast, remained unchanged across all cell lines (p = 0.14 and p = 0.34, for G226R and InDel, respectively) (Fig. 6a). In addition, we assessed the intrinsic excitability of iN networks by measuring active and passive membrane properties, including membrane capacitance, AP threshold, AP amplitude, AP half-width, and after hyperpolarisation (AHP). Overall, no significant alterations were observed across these parameters, except for increased AHP in InDel-iNs (p = 0.15 and p = 0.024, for G226R and InDel, respectively) (Supplementary Fig. S4a).

To further validate the significant increase in mEPSC frequency in network cultures, we investigated whether this synaptic phenotype could be recapitulated in murine hippocampal network cultures. Neurons were transduced with a lentivirus expressing either the wild-type or pathogenic human *STX1B* construct. Consistent with the observations in iN networks, analysis of mEPSCs in the murine hippocampal cultures revealed a significantly increased frequency in G226R-expressing (but not InDel-expressing) neurons compared to those overexpressing wild-type *STX1B* (p = 0.003 and p > 0.99, for G226R and InDel, respectively) (Fig. 6b), while amplitudes remained unaffected (p > 0.99 and p > 0.99, for G226R and InDel, respectively).

Given the observed differences in mEPSC frequency in both iN and murine hippocampal network cultures, we next investigated whether these synaptic phenotypes could influence network-level activity. We utilised multiwell MEAs, on which we co-cultured human iPSC-derived neurons with mouse astrocytes for up to six weeks. Network activity was recorded weekly from DIV 28 to DIV 42. Neurons carrying one of the *STX1B* variants exhibited an overall increased network activity when compared to control iN networks (Fig. 6d). G226R-iNs showed a significant increase in the total number of spikes compared to both controls and InDel-iNs (p < 0.0001, p = 0.90 and p < 0.0001, for G226R vs Control, InDel vs Control and G226R vs InDel, respectively) (Fig. 6c). To further investigate the nature of these spikes, we distinguished between burst-unrelated spikes (interburst spikes) and those occurring within bursts (intraburst spikes) (Supplementary Fig. S4b). This indicates a variant-specific difference in interburst spiking, with G226R-iNs showing a significant increase, whereas InDel-iNs exhibit a marked reduction (p < 0.0001, p = 0.006 and p < 0.0001, for G226R vs Control, InDel vs Control and G226R vs InDel, respectively) (Fig. 6c). Next, we assessed global synchronous network activity by quantifying network bursts (NB), which were detected by simultaneous burst activity across a minimum of 6 out of 12 electrodes. iN networks containing either of the two *STX1B* variants displayed a significant increase in NB number (p < 0.0001, p = 0.004 and p = 0.001, for G226R vs Control, InDel vs Control and G226R vs InDel, respectively) (Fig. 6c) and NB duration (p < 0.0001, p = 0.049 and p = 0.17, for G226R vs Control, InDel vs Control and G226R vs InDel, respectively) (Fig. 6e) compared to control neurons, thus revealing a shared burst pattern across both variants which was more pronounced for G226R. Interestingly, although burst duration was prolonged, the number of intraburst spikes remained comparable to that of control neurons, resulting in a significantly reduced NB spike frequency in both variants (p < 0.0001, p < 0.001 and p > 0.99, for G226R vs Control, InDel vs Control and G226R vs InDel, respectively) (Fig. 6e). This bursting pattern was not restricted to synchronous network activity, but was also observed in isolated bursts outside of network events (Supplementary Fig. S4c).

To investigate whether network activity profiles could stratify the different *STX1B* genotypes, we performed a PCA incorporating seven distinct parameters including total spike count, interburst spike ratio, NB count per minute, NB duration, NB inter-burst interval, NB spike frequency, and the coefficient of variation of the NB inter-burst interval. This analysis revealed separation of both *STX1B* variants from the control group, with the G226R variant showing the most pronounced divergence (Fig. 6f ).

Overall, the DEE-associated G226R variant exhibited a much more pronounced network phenotype compared to the GEFS + -linked InDel variant, suggesting that the severity of the clinical presentation is mirrored by the extent of network dysregulation in iNs.

To also capture potential changes in the temporal dynamics during development of the network activity, we analysed the time course of the network's spiking and bursting activity. Network activity remained largely stable between DIV 28 and DIV 42. G226R-iN networks showed only minor variations during this period, whereas InDel-iN networks exhibited a modest increase, most evident in the total spike count (Supplementary Fig. S4d).

### Bulk RNA sequencing identifies a transcriptomic signature in patient-derived iNs

**Fig. 7:**
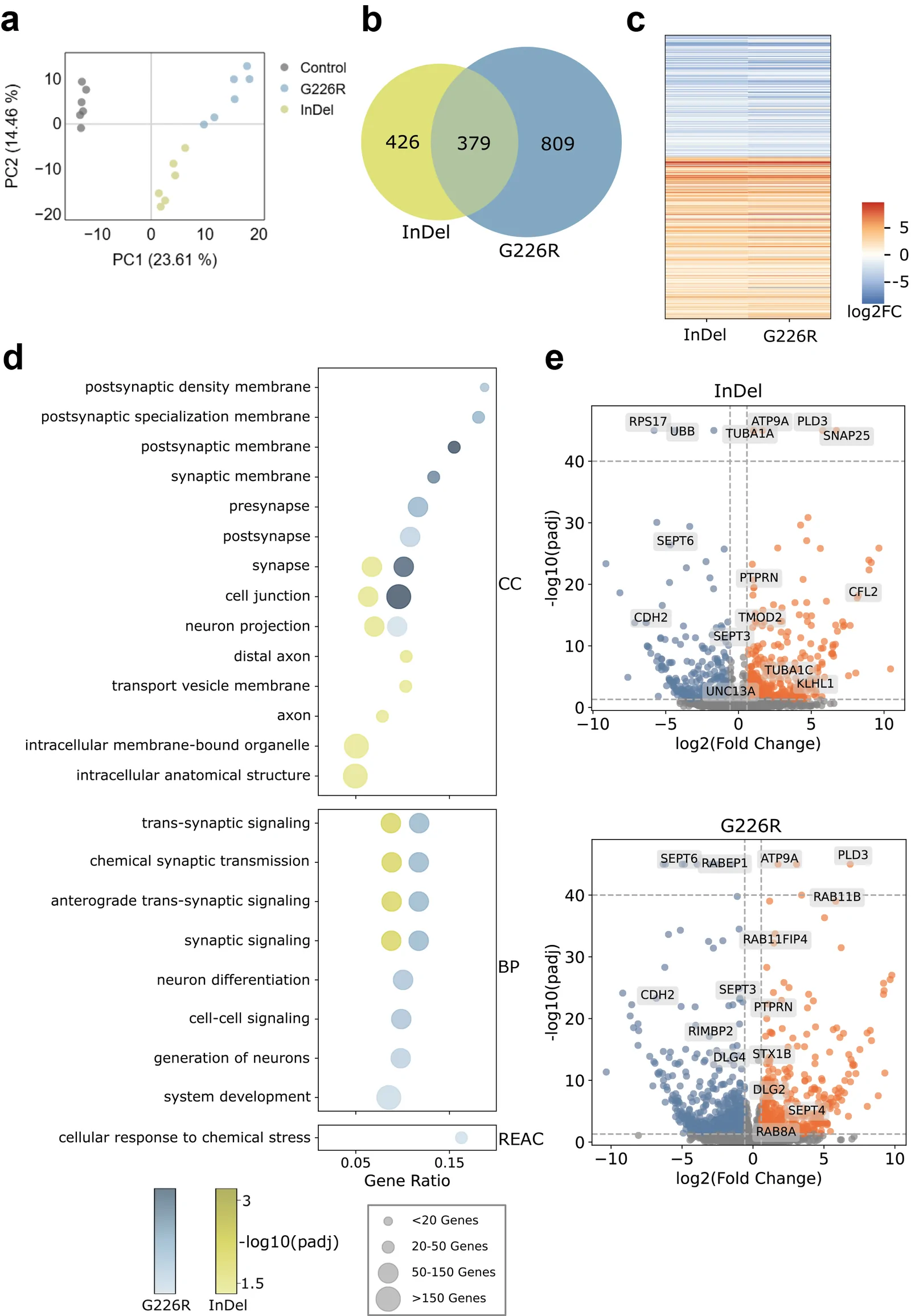
**Transcriptomic profiling reveals shared and divergent gene expression changes in patient-derived iNs**. **a** The PCA demonstrates a clear separation between iNs carrying the G226R or InDel *STX1B* variants and control iNs, indicating distinct transcriptional signatures. **b** Venn diagram highlighting the overlap of 379 DEGs that are commonly altered in both G226R and InDel patient-derived neurons. **c** Heatmap displaying the log_2_ fold changes of the shared DEGs, highlighting consistent expression patterns across variants. **d** Bubble plot summarising enriched gene ontology (GO) terms across categories, including cellular component (CC), biological process (BP), and reactome (REAC) pathways, revealing functional themes associated with the DEGs. **e** Volcano plots depicting the transcriptomic profiles of InDel (top panel) and G226R (bottom panel) iNs. Genes significantly upregulated or downregulated (log_2_FC > |0.58|, adjusted p < 0.05) are highlighted in orange and blue, respectively. RNA isolation and processing were performed from three independent differentiations per cell line. Abbreviations: FC, fold change; PC, principal component.

To capture transcriptomic changes linked to *STX1B* variants, bulk RNA sequencing was conducted on iN co-cultures at DIV 28. PCA revealed a clustering of controls, pooled from two independent individuals with distinct genetic backgrounds, and a clear segregation of the patient-derived lines G226R and InDel (Fig. 7a). The first two principal components explained 23.6% and 14.5% of the total variance, respectively, contributing to a combined variance of 38.1%, and suggesting meaningful transcriptomic differences between the groups.

Gene expression analysis identified 1188 and 805 differentially expressed genes (DEGs) in G226R and InDel, respectively (adjusted p < 0.05, |log_2_FC| > 0.58). In both variants, the proportions of up- and downregulated genes were comparable. A substantial overlap of 379 DEGs was observed between G226R and InDel (Fig. 7b), with the majority of shared DEGs exhibiting a concordant direction of regulation (Fig. 7c), indicating potential convergent molecular mechanisms.

GO enrichment analysis revealed a significant overrepresentation of terms associated with synaptic structure and function, including “synapse”, “cell junction”, and “neuron projection” (Fig. 7d). Biological process categories predominantly reflected alterations in synaptic signalling pathways.

In addition to shared features, each variant exhibited distinct transcriptomic signatures. InDel-specific DEGs were enriched for genes involved in cytoskeletal organisation and intracellular architecture (e.g., *SEPT6*, *SEPT3*, *CFL2*, *TMOD2*, *KLHL1*, *TUBA1A*, *TUBA1C*), suggesting alterations in neuronal cytoarchitecture (Fig. 7e, top panel). Notably, analysis of SNARE-related genes revealed upregulation of *SNAP25* and a subtle downregulation of *UNC13A*, encoding MUNC13-1, a key regulator of SNARE complex assembly.

G226R-iNs displayed pronounced transcriptional changes indicative of structural remodelling at both pre- and postsynaptic sites. Upregulation of *STX1B* was accompanied by reduced expression of *RIMBP2*, a critical component of the presynaptic active zone involved in coupling voltage-gated calcium channels to synaptic release sites (Fig. 7e, bottom panel). Moreover, altered expression of postsynaptic scaffold proteins *DLG4* (PSD-95) and *DLG2* (PSD-93) suggests postsynaptic reorganisation. A notable increase in dysregulated genes related to RAB GTPases was observed, including *RAB11*, *RAB8A*, *RAB31*, as well as RAB-interacting and regulatory proteins such as *ATP9A*, *RAB11FIP4*, and *RABEP1*. These findings implicate altered RAB-associated vesicular trafficking dynamics in patient-derived iNs.

Taken together, patient-derived iNs exhibit partially overlapping transcriptomic changes, affecting genes implicated in synaptic structure, signalling, and vesicle trafficking.

## Discussion

The present study functionally characterised *STX1B* variants in a human iPSC-based neuronal system derived from patient-specific cells. Prior work in mouse neurons demonstrated that these variants can disrupt synaptic function under an unphysiological condition lacking both *Stx1a* and *Stx1b*.^20^ In this previous model, InDel-expressing neurons represented a complete LOF due to reduced protein stability, while G226R displayed a milder LOF effect on Ca^2+^-triggered neurotransmitter release. However, since neither of the two variants exhibited a phenotype in the presence of wild-type syntaxin-1B and syntaxin-1A, their impact on synaptic function in a heterozygous state remains to be determined. Addressing this gap, this study investigated the variant effects in a human genetic context, enabling the analysis of disease-relevant mechanisms under conditions that more closely resemble those in affected individuals.

Building on this human model, our electrophysiological analyses revealed that both *STX1B* variants under investigation were associated with a hyperexcitable network state in *NGN2*-induced excitatory neuronal cultures. Using MEA recordings, we observed significantly increased burst frequency and length in iN networks derived from affected individuals compared to those from controls, suggesting a shared network-level phenotype despite the distinct molecular nature of the variants. Notably, this hyperactivity was significantly more pronounced in G226R-iN networks, aligning with the variants’ association with more severe clinical manifestations. Whole-cell patch-clamp recordings further demonstrated that only G226R produced a measurable electrophysiological phenotype at the synaptic level. Specifically, G226R showed an increased mEPSC frequency as a network-specific phenotype, along with a failure to maintain neurotransmission during sustained high-frequency activity. Importantly, passive and active membrane properties in iN networks were largely unchanged across variants, arguing against cell-autonomous alterations as the basis of the network phenotype. Although an increased AHP was observed in InDel neurons, this isolated effect does not readily explain the observed network hyperexcitability, suggesting a predominantly synaptic origin.

Despite its location within the highly conserved SNARE motif,^19^ the G226R variant does not impair syntaxin-1B's ability to assemble into SNARE complexes, as shown by prior *in vitro* reconstitution assays and structural investigations.^20^ However, it appears to interfere with interactions involving other key regulatory proteins of the synaptic vesicle release machinery. Biochemical studies have shown that the binding affinity of syntaxin-1B G226R for Munc18-1 is strongly reduced, likely due to electrostatic repulsion between the inserted arginine residue and the positively charged cavity of the Munc18-1 binding interface (Supplementary Fig. S5a).^20^ This impaired interaction may compromise two essential functions of Munc18-1: first, its role in stabilising syntaxin-1B in the closed conformation, and second, its ability to assist in the proper alignment of the opened syntaxin-1B with synaptobrevin and SNAP25 during Munc13-1-mediated priming.^1^^,^^5^^,^^44^ This alignment intermediate state represents a critical checkpoint in SNARE complex assembly. Consequently, the reduced Munc18-1 interaction may act as a rate-limiting step in vesicle priming. Functionally, this molecular perturbation may underlie the synaptic phenotype observed in G226R-iNs. In the present study, we found that while initial vesicle priming and RRP size were only mildly affected during single-pulse stimulation, synaptic depression was markedly exacerbated during sustained high-frequency stimulation. These findings suggest that the impaired interaction with Munc18-1 becomes functionally limiting under the demanding conditions of sustained high-frequency neurotransmission, when rapid vesicle priming is needed to replenish the RRP. As RRP replenishment depends on the continuous supply of primed vesicles,^45^ the reduced recycling capacity in G226R-iNs likely reflects a bottleneck at this step, ultimately resulting in diminished charge transfer and reduced synaptic fidelity during ongoing activity.

In addition to this activity-dependent synaptic fatigue, G226R-iNs exhibited a significantly increased frequency of mEPSCs. One plausible mechanism underlying this gain-of-function phenotype is interference of the variant with the clamping activity of synaptotagmin-1. Under basal conditions, the SNARE complex must be tightly regulated to prevent premature fusion, with synaptotagmin-1 acting as a calcium-sensitive clamp that stabilises vesicles in a release-ready yet restrained state.^46^^,^^47^ This clamp is based on electrostatic interactions between basic residues of synaptotagmin-1 (R281, K288, R398, R399) and acidic residues on syntaxin-1B (D230, E233, E237) and SNAP25 (D51, E52, E55) (Supplementary Fig. S5b).^48^ The G226 residue lies in close proximity to this interface, and its substitution with a bulky, positively charged arginine may alter the local electrostatic environment, potentially weakening synaptotagmin-1 binding. Such a disruption has the potential to destabilise the clamped intermediate state and increase the likelihood of spontaneous vesicle fusion.^49^

Of note, the increased frequency of mEPSCs in G226R was observed in iN–as well as mouse-network cultures but not in autaptic cultures. This divergence between network and autaptic cultures exemplifies a well-known phenomenon where synaptic phenotypes are often subtle or absent in isolated single neurons yet become apparent within the more physiologically relevant context of interconnected networks.^50^, ^51^, ^52^ Although the exact reasons remain unclear, it is generally thought that the complex synaptic connectivity and interactions present in networks are essential for unmasking these functional alterations.

In contrast to G226R, the complex InDel variant is located within the first helix of the H_abc_-domain.^19^ The iPSC lines used in this study were derived from two related individuals with GEFS + who experienced a mild form of epilepsy that resolved during childhood without further treatment. This clinical presentation aligns with the network-level hyperactivity observed in InDel-derived cultures, which, however, did not translate into detectable synaptic alterations at the single-cell level.

Structurally, the deletion of K45 combined with the insertion of five additional amino acids and the L46M substitution disrupts syntaxin-1B protein stability and leads to a non-functional protein in autapses of *Stx1a* and *Stx1b* knock-out mice.^20^ In line with these results, protein quantification by Western blot showed reduced levels of syntaxin-1B in InDel-iNs. Prior studies in *Stx1b* knockout mice reported a smaller RRP size, decreased vesicular release probability, and reduced mEPSC frequency.^14^^,^^15^^,^^53^ These findings are indicative of a LOF phenotype that would be anticipated in the context of reduced syntaxin-1B protein. In the present study, however, InDel-iNs did not show comparable deficits in single-cell electrophysiology. Instead, synaptic density was markedly increased, reaching more than twice the level of controls, while the strength of synaptic responses remained unchanged. Given the established inverse relationship between synapse number and release probability,^54^ this likely reflects a homoeostatic adjustment to compensate for impaired release efficiency and may effectively mask the expected LOF phenotype.

The higher synaptic density observed in InDel-iNs additionally corresponds with changes in the expression of genes related to cytoskeletal dynamics and neuronal morphology. Notably, these neurons also show a strong upregulation of the SNARE protein SNAP25. While SNAP25 cannot simply replace the function of syntaxin-1B,^55^ its elevated expression may enhance the binding efficiency of the remaining syntaxin-1B protein. The syntaxin-1-SNAP25 dimer is a critical and rate-limiting intermediate in SNARE assembly^56^ and also supports neuronal survival independently of neurotransmission.^57^ Therefore, elevated levels of SNAP25 could increase the probability of effective interactions with residual wild-type syntaxin-1B, helping to preserve synaptic output in isolated neurons while promoting aberrant network-level excitability.

Although neurons containing either InDel or G226R share a remarkable overlap in synapse-related differentially expressed genes, G226R-iNs also exhibit distinct transcriptional changes. The upregulation of *STX1B* in G226R-iNs, despite normal protein abundance, may reflect increased protein turnover driven by the gain-of-function phenotype: elevated spontaneous vesicle fusion increases SNARE complex consumption, while the reduced Munc18-1 interaction may render syntaxin-1B more susceptible to degradation in its unbound state, collectively necessitating higher transcriptional output to maintain steady-state protein levels. Notably, although InDel-iNs present with reduced syntaxin-1B protein (a condition that would equally warrant transcriptional upregulation), these neurons appear to engage alternative compensatory mechanisms, including increased synaptic density and *SNAP25* upregulation, rather than elevating *STX1B* expression itself. Additionally, downregulation of *RIMBP2*, a key active zone organiser involved in vesicle tethering and replenishment,^58^ may correlate with the impairment in synaptic reliability. Concomitant dysregulation of RAB-associated genes suggests broader disruption of vesicle trafficking and recycling pathways.^59^ The observed downregulation of DLG4 (PSD-95), together with upregulation of DLG2 and reduced expression of the AMPA receptor auxiliary subunit SHISA6, points towards postsynaptic remodelling with potentially altered receptor stabilisation and synaptic organisation. Notably, SHISA6 has been shown to prevent synaptically trapped AMPARs from depression during high-frequency synaptic transmission,^60^ suggesting that its reduced expression may further compromise postsynaptic responsiveness under sustained activity. Together, these changes may represent a molecular correlate of the mixed loss- and gain-of-function phenotype, characterised by increased spontaneous excitatory activity in networks but impaired synaptic reliability during sustained high-frequency stimulation.

Taken together, these findings suggest that the increased network activity observed in InDel and G226R neurons may arise from partially convergent, compensatory mechanisms that are initially triggered by distinct primary synaptic dysfunctions. G226R appears to affect synaptic transmission through LOF mechanisms, such as synaptic failure during sustained high-frequency activity, and GOF effects, including enhanced spontaneous vesicle release. In contrast, the InDel variant primarily reduces syntaxin-1B protein levels, reflecting a classical LOF phenotype. In both cases, transcriptomic adaptations engage homoeostatic mechanisms aimed at stabilising synaptic function, yet these compensatory changes may paradoxically increase susceptibility to network hyperexcitability and contribute to disease-relevant neuronal phenotypes.

It is important to consider that the described compensatory mechanisms were observed in a purely excitatory *NGN2*-derived neuronal network, which not only lacks inhibitory neurons but also the broader cell-type diversity found *in vivo*. Therefore, this setup likely triggers strong homoeostatic responses that may not accurately represent the complex dynamics of the human brain. Furthermore, the model does not reflect synaptic heterogeneity or cell type-specific features, which are crucial for understanding how variants differently affect synapse subtypes. For example, the G226R variant may particularly impact synapses involved in high-frequency neurotransmission, such as those of fast-spiking interneurons. These cell type-specific vulnerabilities can disrupt the delicate balance between excitation and inhibition. In addition, *NGN2*-induced neurons are known to exhibit substantial molecular and functional heterogeneity, generating diverse neuronal subtypes and maturation states depending on factors such as expression levels and differentiation conditions.^61^ This intrinsic variability may further influence the observed electrophysiological phenotypes and should be considered when interpreting network-level effects.

Another limitation of this study is the absence of direct causal validation linking the observed phenotypes to the *STX1B* variants, as rescue experiments through the generation of isogenic controls were not performed. While the use of patient-derived iPSCs provides a relevant genetic context, the contribution of genetic background effects cannot be fully excluded. Future studies incorporating isogenic rescue approaches could further strengthen the causal interpretation of the observed phenotypes.

Despite these considerations, our findings represent an important piece in dissecting SNARE-related pathologies in a human model representing the genetic constellation of affected individuals and underscore the need for future investigations using more sophisticated models to fully elucidate variant-specific effects on neuronal networks.

## Contributors

The study was designed by CH, NS, and HL. HM, BK, and HL obtained patient material. CH performed and analysed all iPSC-related experiments with support from FS, FK, BU, FG, OV, HLÖ, JC, KB, and NS. Mouse experiments were performed and analysed by YL. MBO conducted the data analysis of bulk RNA sequencing. CH, NS, RFT, and HL interpreted the data. CH and NS accessed and verified the underlying data. CH wrote the original draft, which was edited by NS, RFT and HL. All authors read and approved the final version.

## Data sharing statement

The bulk RNA sequencing dataset generated during this study has been deposited in NCBI's Gene Expression Omnibus and is publicly accessible through the GEO Series accession number GSE329848.

All other de-identified quantitative datasets underlying the findings of this study, including single-cell patch-clamp recordings, multi-electrode array (MEA) data metrics, and immunofluorescence quantification matrices, are available from the corresponding authors upon reasonable request for the purpose of scientific replication.

## Declaration of interests

HL reports the following disclosures within the past 36 months: research support from Lario Therapeutics. He has received consulting fees from Praxis Medicine (personal payments) and from Lario Therapeutics (payments made to the University of Tübingen). He has served on a safety monitoring board for Intrabio and has participated in grant review and funding advisory activities for Telethon Italy. All other authors declare no competing interests.
