# Supplementary material for: STX1B variant-specific synaptic dysfunction is associated with network hyperexcitability in human iPSC-derived neurons
Source: eBioMedicine. 2026 Aug 31;131:106451. doi: 10.1016/j.ebiom.2026.106451 (PMC13560554; doi:10.1016/j.ebiom.2026.106451)
Supplement: Supplementary file 1 [file mmc1.pdf]

Supplementary Fig. S1: Characterisation and Validation of iPSC Lines.

a Karyotyping of all iPSC lines used in this study confirmed normal chromosomal integrity, with no detectable structural abnormalities.
b Immunocytochemical staining for pluripotency markers OCT4, TRA-1-60, SOX2, and SSEA-4 demonstrates robust expression of key pluripotency factors in all newly generated iPSC lines. Scale bar: 200 µm.

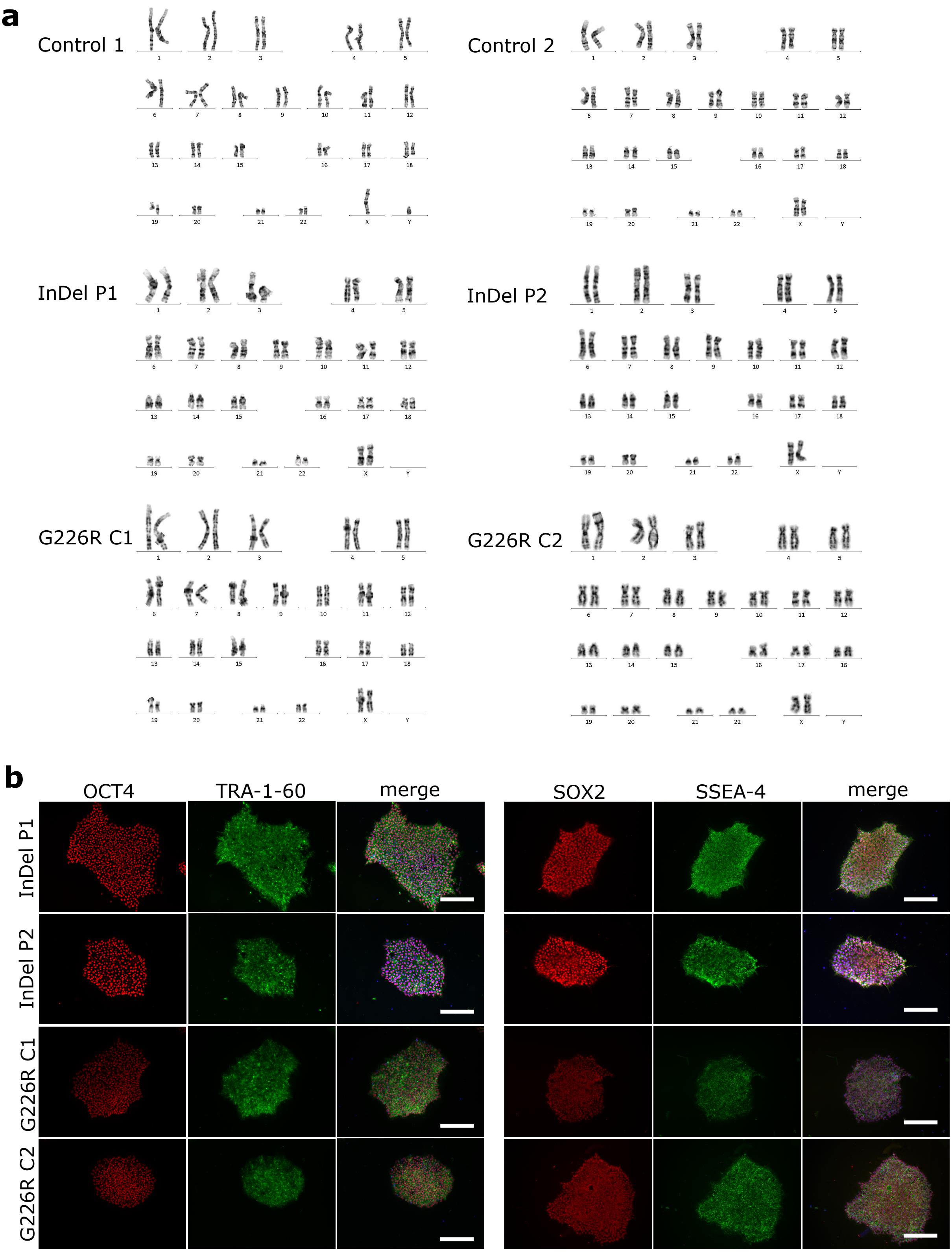
a
Control 1
Control 2
InDel P1
InDel P2
G226R C1
G226R C2
1 2 3 4 5 6 7 8 9 10 11 12 13 14 15 16 17 18 19 20 21 22 X Y
b
OCT4
TRA-1-60
merge
SOX2
SSEA-4
merge
InDel P1
InDel P2
G226R C1
G226R C2

Supplementary Fig. S2: Short-Term Synaptic Plasticity of Autaptic iNs.

a Normalised synaptic responses plotted as a function of pulse number during 10 Hz (left) and 20 Hz (right) stimulation trains. Both *STX1B* variants exhibit a use-dependent increase in synaptic depression, which is particularly pronounced at 20 Hz.
b Analysis of paired-pulse ratios across varying inter-pulse intervals (50 ms, 100 ms, 200 ms and 1000 ms) revealed comparable responses across groups, with no significant differences detected. Data are visualised as boxplots, where whiskers extend to 1·5 times the interquartile range, the solid line marks the median, and the dashed line indicates the mean. Data are based on four independent differentiations. n = 31-37 (control), 28-37 (G226R) and 25-36 (InDel). Statistical comparisons in **a** were done using permutation-based mixed-design ANOVA followed by post hoc permutation-based independent t-test with Holm's multiple comparison correction, in **b** were carried out using a Kruskal–Wallis test followed by Dunn's multiple comparisons post hoc test; Abbreviations: n.s., not significant.

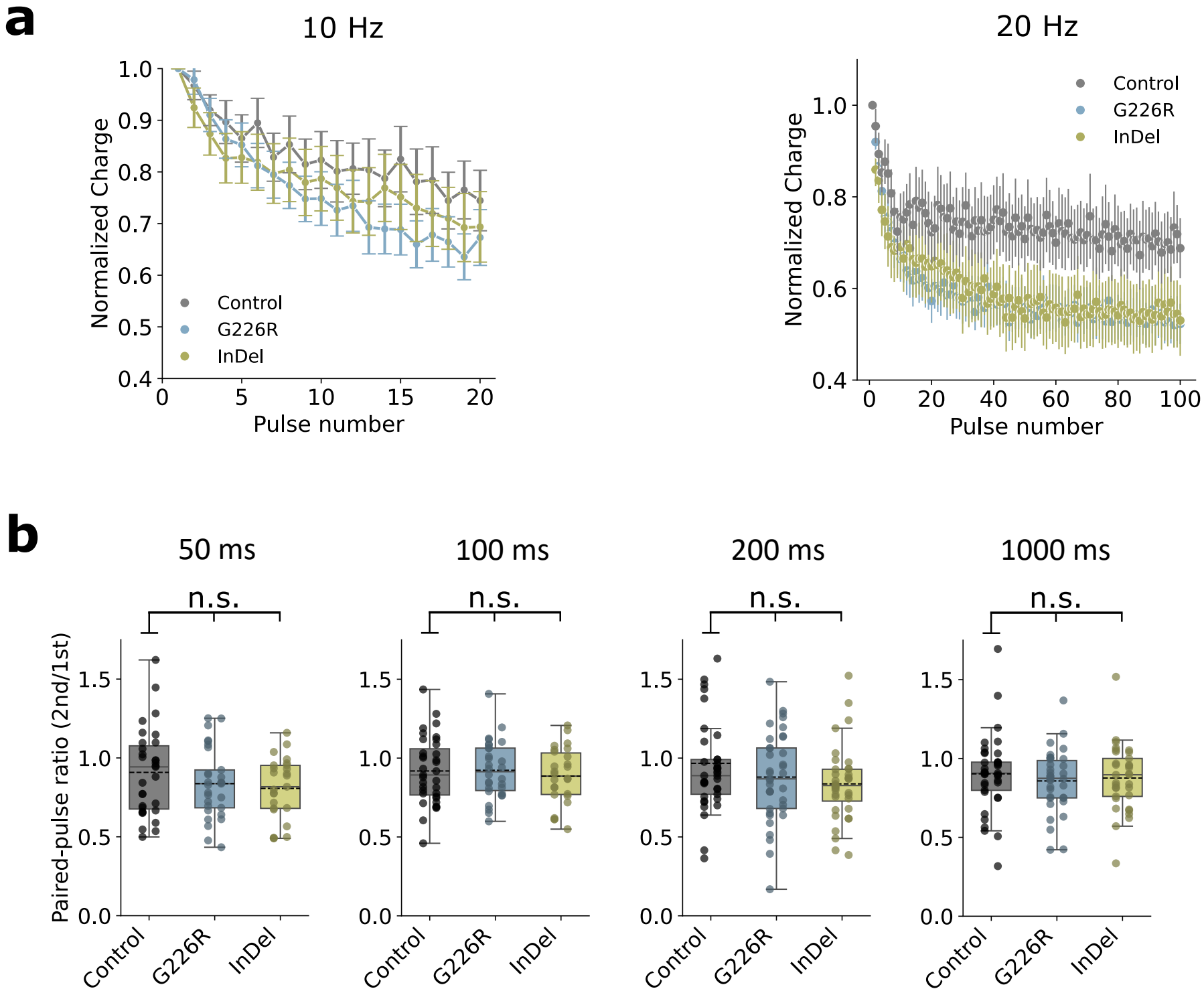

a
10 Hz
20 Hz
Normalized Charge
Pulse number
Control
G226R
InDel
b
50 ms
100 ms
200 ms
1000 ms
n.s.
Paired-pulse ratio (2nd/1st)
Control
G226R
InDel

Supplementary Fig. S3: Colocalisation of the presynaptic marker Bassoon and the postsynaptic marker Homer indicates functional synapses in iN-autaptic and network cultures.

a Representative immunofluorescence images showing MAP2-labelled autaptic neurons and synaptic structures marked by the presynaptic protein bassoon and the postsynaptic protein homer (Scale bars: 50 µm for overview images; 5 µm for the bassoon (red) / homer (green) colocalisation magnification).
b Representative immunofluorescence images of MAP2-labelled neurons in iN network cultures, illustrating presynaptic bassoon and postsynaptic homer puncta (Scale bars: 50 µm for overview images; 5 µm for the bassoon (red) / homer (green) colocalisation magnification).
c Quantification of functional synapses in iN networks based on bassoon/homer colocalisation. Synapse density was quantified as the number of bassoon/homer colocalisations per 100 µm of dendrite length, showing a significant increase in InDel-derived neurons. Data are visualised as boxplots, with whiskers indicating the range within 1·5× the interquartile range. Solid lines mark the median, and dashed lines indicate the mean. Each dot represents a single image. Data are based on four independent differentiations. n = 27 (control), 35 (G226R), and 33 (InDel), corresponding to the number of fields of view analysed. Statistical comparisons were performed using the Kruskal–Wallis test followed by Dunn's post hoc analysis; *p < 0·05, **p < 0·01.
Abbreviations: n.s., not significant; Bas, Bassoon; Hom, Homer.

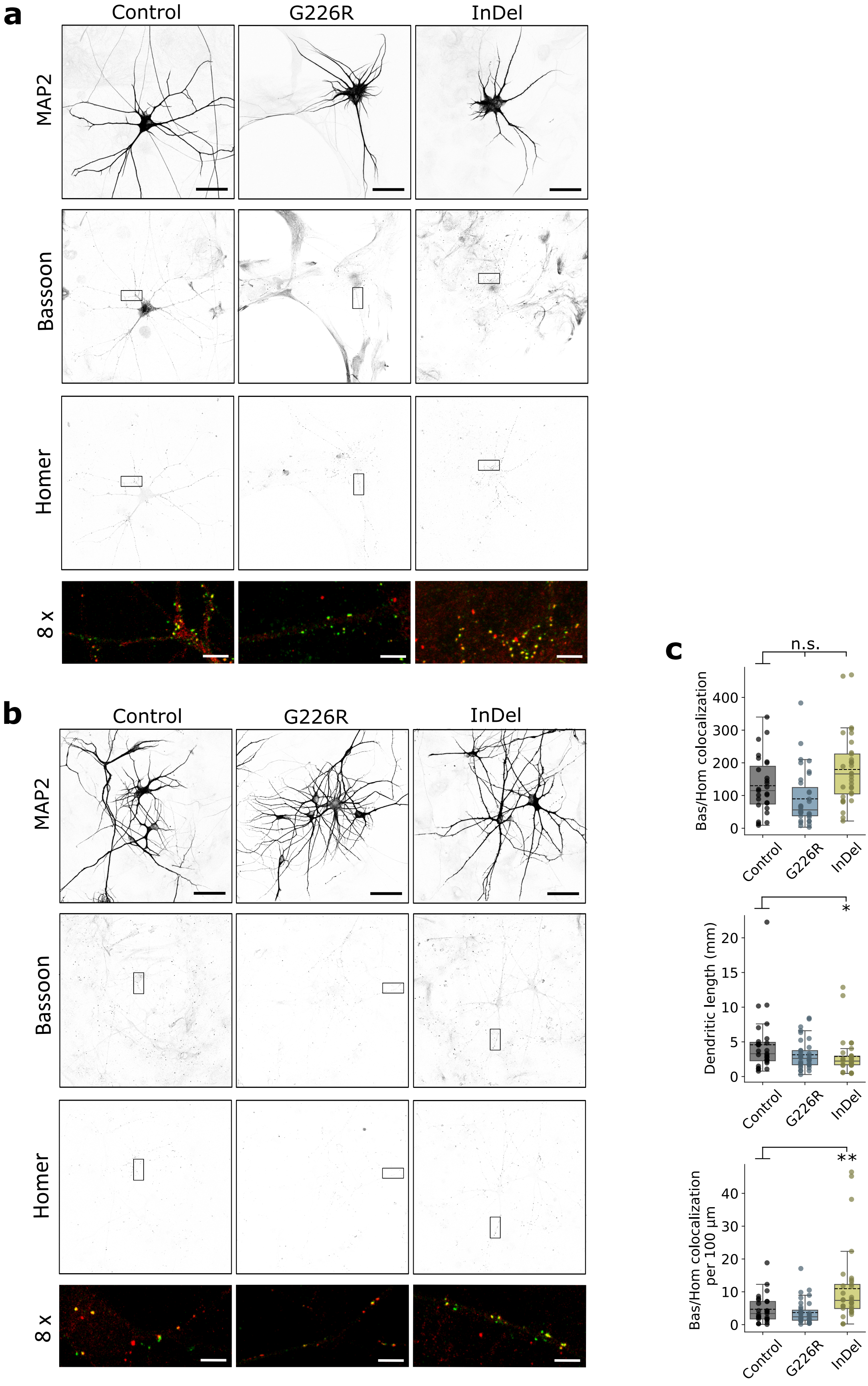

a
Control
G226R
InDel
MAP2
Bassoon
Homer
8 x
b
Control
G226R
InDel
MAP2
Bassoon
Homer
8 x
c
n.s.
Bas/Hom colocalization
400
300
200
100
0
Control
G226R
InDel
*
Dendritic length (mm)
20
15
10
5
0
Control
G226R
InDel
**
Bas/Hom colocalization per 100 µm
40
30
20
10
0
Control
G226R
InDel

Supplementary Fig. S4: Patch-clamp analysis of intrinsic membrane properties and MEA-based temporal network dynamics in iN networks.

a Active and passive membrane properties were assessed by whole-cell patch-clamp recordings of iN networks at DIV35. Analysed parameters include capacitance, AP threshold, AP amplitude, AP halfwidth, and AHP. Data are based on three to four independent differentiations. n = 24 (control), 25 (G226R) and 13 (InDel). Statistical comparisons were carried out using a Kruskal–Wallis test followed by Dunn's multiple comparisons post hoc test; $*p < 0{\cdot}05$.
b Schematic overview of key MEA-derived metrics used to assess neuronal network activity. Created with Biorender.com.
c Burst-specific parameters from multi-well MEA recordings at DIV 28. Both variants displayed an increased burst count, consistent with the pattern observed for network bursts. Burst duration was significantly increased only for G226R. Intra-burst spike count did not differ from control neurons, whereas burst spike frequency was significantly reduced in both variants. Data are shown as boxplots, with whiskers indicating 1·5 times the interquartile range. Solid lines indicate the median and dashed lines indicate the mean. Burst parameters were averaged across all electrodes within a well, and each dot represents one well. Data were obtained from four to eight independent differentiations. n = 89 (control), 65 (G226R) and 38 (InDel). Statistical comparisons were performed using the Kruskal–Wallis test followed by Dunn's post hoc analysis; $*p < 0{\cdot}05$, $**p < 0{\cdot}01$, $***p < 0{\cdot}001$.
d Line plots represent the progression of four MEA parameters (Total Spike Count, Interburst Spike Count, NB/min and Bursts/min) over time, showing mean ± SEM from week 4 (DIV 28) to week 6 (DIV 42). Data are based on four to eight independent differentiations. week 4: n = 89 (control), 65 (G226R) and 38 (InDel); week 5: n = 62 (control), 63 (G226R) and 39 (InDel); week 6: n = 61 (control), 36 (G226R) and 34 (InDel). Statistical comparisons were carried out using a Kruskal–Wallis test followed by Dunn's multiple comparisons post hoc test; $*p < 0{\cdot}05$, $**p < 0{\cdot}01$, $***p < 0{\cdot}001$.
Abbreviations: NB, network burst; DIV, days in vitro; AP, action potential; AHP, afterhyperpolarisation; SEM, Standard error of the mean.

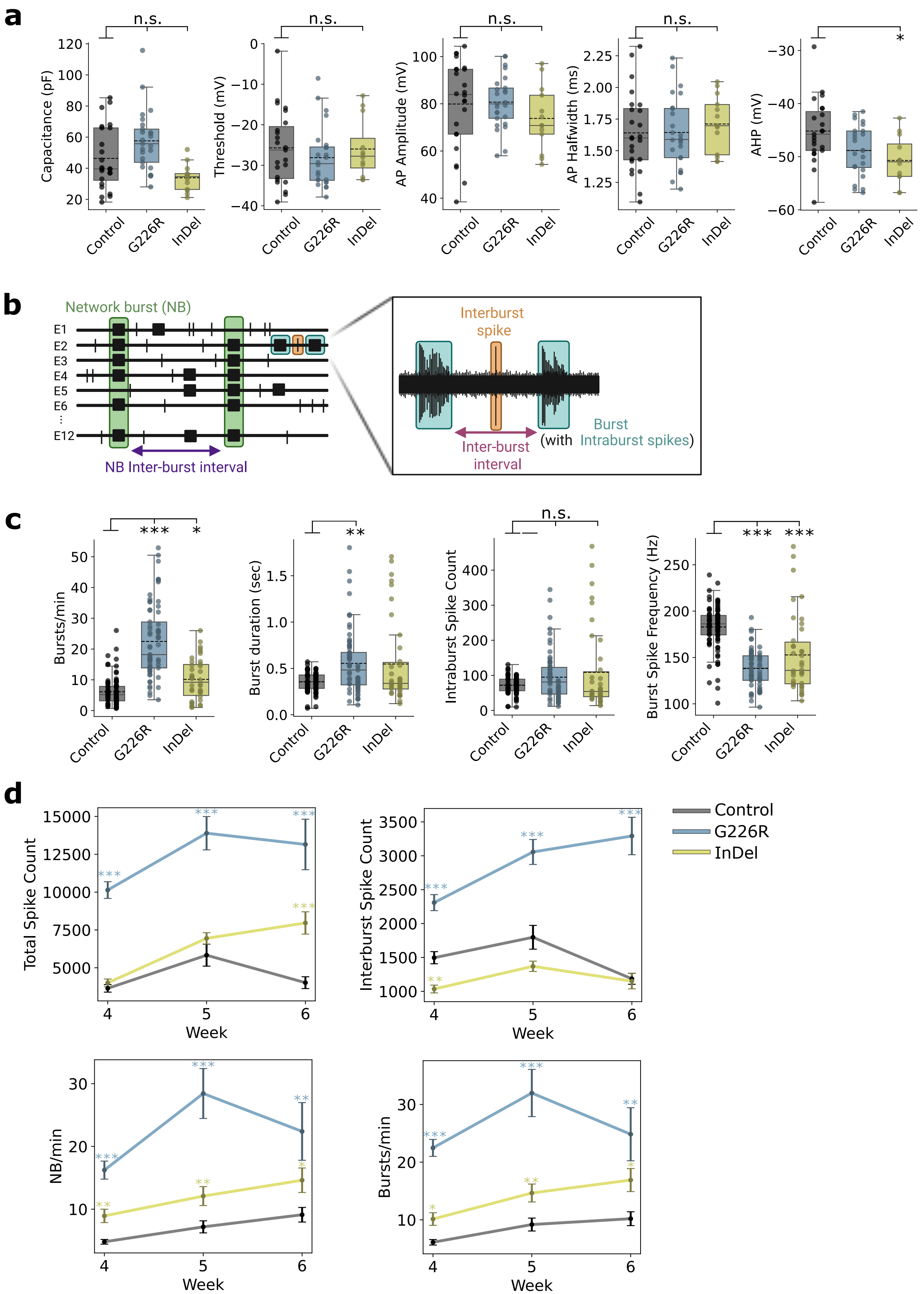

a
n.s.
Capacitance (pF)
Threshold (mV)
AP Amplitude (mV)
AP Halfwidth (ms)
AHP (mV)
*
Control
G226R
InDel
b
Network burst (NB)
E1
E2
E3
E4
E5
E6
E12
NB Inter-burst interval
Interburst spike
Burst
(with Intraburst spikes)
Inter-burst interval
c
Bursts/min
Burst duration (sec)
Intraburst Spike Count
Burst Spike Frequency (Hz)
***
**
d
Total Spike Count
Interburst Spike Count
NB/min
Bursts/min
Week
Control
G226R
InDel

Supplementary Fig. S5: The machinery underlying neurotransmitter release is regulated through specific protein-protein interactions.

a The crystal structure of the MUNC18-1–Syntaxin-1A complex (PDB: 4JEU [1]; *Rattus norvegicus*) shows that the *STX1B* G226R variant (equivalent to G227 in *STX1A*) introduces an arginine residue into a positively charged site of MUNC18-1.

b The crystal structure of the primary interface in the synaptotagmin-1–SNARE complex (PDB: 5CCG [2]; *Rattus norvegicus*) shows that the *STX1B* G226R variant (equivalent to G227 in *STX1A*) is located next to key residues of the primary interface. E238, E234 and D231 in syntaxin-1A corresponds to E237, E233 and D230 in syntaxin-1B, respectively.

The graphics were generated using Mol* Viewer 2.11.1 on www.rcsb.org [3,4]

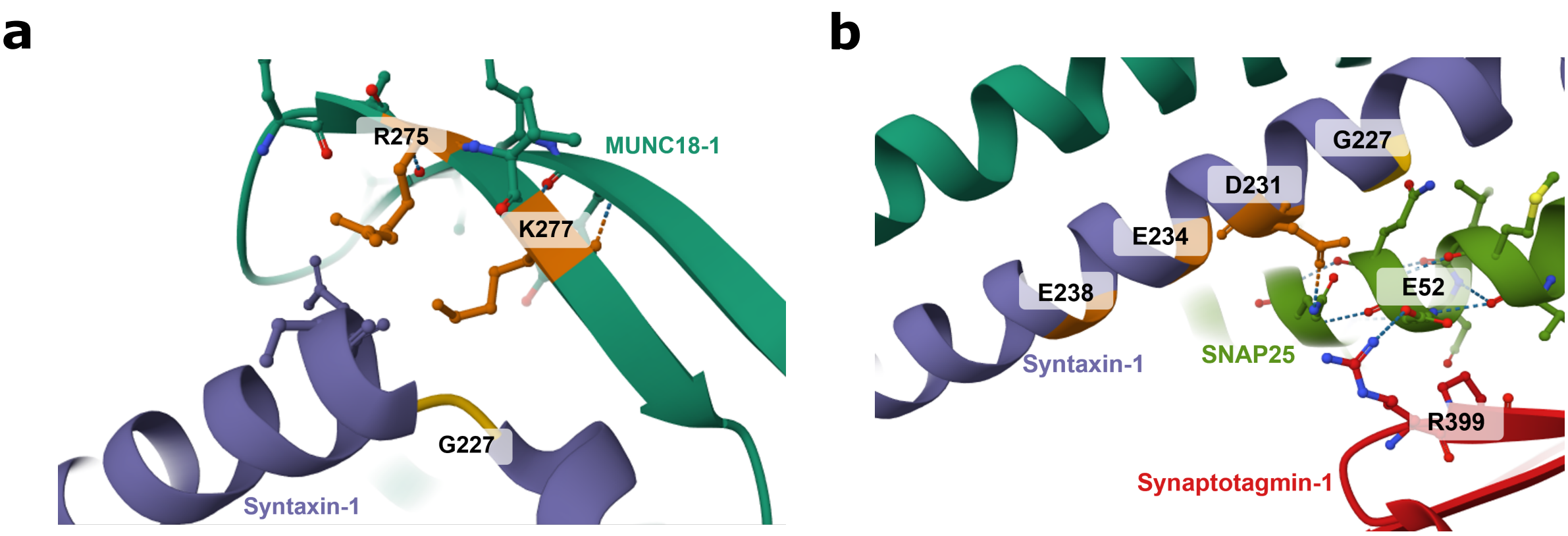
a
R275
K277
MUNC18-1
G227
Syntaxin-1
b
G227
D231
E234
E238
E52
Syntaxin-1
SNAP25
R399
Synaptotagmin-1

Supplementary Tab.1: Summary statistics of results.
Unless otherwise stated, *N/n* indicate the number of independent differentiations and the number of cells, respectively. For MEA data, *n* refers to the number of analysed wells; for Syntaxin-1B level data, *n* refers to the number of cell lysates; for Bassoon/Homer colocalisation data of iN-networks, *n* refers to the number of analysed fields of view. Adjusted *p*-values refer to comparisons between variant and control unless stated otherwise. When two values are reported (denoted as 'value1 / value2'), the first corresponds to the variant vs. control comparison and the second to the comparison between both variants.

Suppl. Table 1

Fig.	Parameter	Statistical test	Group	N/n	Mean ± SEM	Median	adjusted p-value
2b	STX1B/MAP2, Western Blot	Kruskal-Wallis	Ctrl	4/8	1 ± 0·315	0·64	
			G226R	4/7	0·61 ± 0·151	0·36	0·3961
			InDel	4/8	0·34 ± 0·063	0·37	0·0219
2c	MAP2/Vinculin, Western Blot	Kruskal-Wallis	Ctrl	4/8	1 ± 0·259	0·78	
			G226R	4/7	2·596 ± 1·076	1·37	0·4105
			InDel	4/8	1·470 ± 0·327	1·24	0·3940
3b	mEPSC frequency, autaptic	Kruskal-Wallis	Ctrl	4/35	0·26 ± 0·05	0·13	
			G226R	4/36	0·26 ± 0·04	0·19	0·5271
			InDel	4/33	0·14 ± 0·04	0·07	0·0580
3b	mEPSC amplitude, autaptic	Kruskal-Wallis	Ctrl	4/32	58·29 ± 4·24	54·78	

			G226R	4/35	42·79 ± 2·10	40·23	0·0112
			InDel	4/32	45·09 ± 4·23	40·94	0·0167
3d	EPSC amplitude, autaptic	Kruskal-Wallis	Ctrl	4/37	1·86 ± 0·20	1·76	
			G226R	4/40	1·72 ± 0·25	1·05	0·4672
			InDel	4/41	1·74 ± 0·31	1·37	0·4504
3d	EPSC charge, autaptic	Kruskal-Wallis	Ctrl	4/37	14·77 ± 1·49	13·96	
			G226R	4/40	10·92 ± 1·24	8·96	0·0930
			InDel	4/41	12·33 ± 1·73	10·54	0·1701
3e	Charge 10 Hz, autaptic	Permutation-based mixed-design ANOVA/ Posthoc Permutation ind. ttest	Ctrl	4/35			
			G226R	4/29			0·9767
			InDel	4/26			0·9767
3e	Cumulative charge, 10 Hz, 20 pulses	Kruskal-Wallis	Ctrl	4/35	244·2 ± 25·58	205·4	
			G226R	4/29	211·6 ± 26·20	184·0	0·6045

			InDel	4/26	252·8 ± 32·83	241·1	>0·9999
3f	Charge 20 Hz, autaptic	Permutation-based mixed-design ANOVA/ Posthoc Permutation ind. ttest	Ctrl	4/31			
			G226R	4/28			0·0108
			InDel	4/25			0·7947
3f	Cumulative charge, 20 Hz, 20 pulses	Kruskal-Wallis	Ctrl	4/31	225·5 ± 27·20	189·4	
			G226R	4/28	144·0 ± 18·00	117·9	0·0382
			InDel	4/25	234·4 ± 34·39	200·2	>0·9999
4a	RRP estimate, autaptic	Kruskal-Wallis	Ctrl	4/21	206·9 ± 50·08	105·7	
			G226R	4/21	122·4 ± 16·68	91·33	0·9117
			InDel	4/17	133·5 ± 23·41	117·2	>0·9999
4b	Release probability, autaptic	Kruskal-Wallis	Ctrl	4/21	12·07 ± 1·5	12·92	
			G226R	4/21	11·73 ± 1·6	9·30	>0·9999
			InDel	4/17	16·62 ± 2·3	16·81	0·3244

4c	EPSC recovery, autaptic	Kruskal-Wallis	Ctrl	4/35	61·56 ± 3·2	59·85	
			G226R	4/29	50·20 ± 3·1	49·11	0·0287
			InDel	4/26	53·34 ± 3·7	52·93	0·2396
4e	Vesicle recruitment rate, autaptic	Kruskal-Wallis	Ctrl	4/31	9·63 ± 1·46	8·36	
			G226R	4/28	5·26 ± 0·7	3·99	0·0201
			InDel	4/25	8·69 ± 1·5	7·34	>0·9999
5b	Synapsin puncta, autaptic	Kruskal-Wallis	Ctrl	4/36	145·2 ± 20·6	99·5	
			G226R	4/42	109·5 ± 21·0	64·0	0·2261
			InDel	4/38	372·0 ± 37·5	390·5	0·0002
5c	Dendritic length, autaptic	Kruskal-Wallis	Ctrl	4/36	1·90 ± 0·17	1·79	
			G226R	4/42	1·94 ± 0·28	1·47	0·5482
			InDel	4/38	1·28 ± 0·13	1·06	0·0109
5d	Synaptic density, autaptic	Kruskal-Wallis	Ctrl	4/36	10·46 ± 1·92	6·56	
			G226R	4/42	8·80 ± 1·97	4·50	0·5446

			InDel	4/38	44·15 ± 8·09	29·33	<0·0001
6a	mEPSC frequency, network	Kruskal-Wallis	Ctrl	3/22	0·22 ± 0·06	0·17	
			G226R	3/28	0·83 ± 0·11	0·68	<0·0001
			InDel	3/19	0·17 ± 0·07	0·08	0·5626
6a	mEPSC amplitude, network	Kruskal-Wallis	Ctrl	3/22	23·21 ± 2·05	21·46	
			G226R	3/28	26·96 ± 1·30	25·13	0·1389
			InDel	3/19	27·39 ± 3·11	24·76	0·3378
6b	mEPSC frequency, mouse	Kruskal-Wallis	Ctrl	-/12	4·07 ± 0·77	3·06	
			G226R	-/11	9·94 ± 0·95	10·70	0·0025
			InDel	-/10	3·16 ± 0·41	2·86	>0·9999
6b	mEPSC amplitude, mouse	Kruskal-Wallis	Ctrl	-/12	23·64 ± 2·24	21·71	
			G226R	-/11	24·03 ± 1·13	25·30	>0·9999
			InDel	-/10	21·77 ± 2·14	21·64	>0·9999
6c / S4d	Total spike count, DIV 28, MEA	Kruskal-Wallis	Ctrl	8/89	3640 ± 248·8	3241	

			G226R	4/65	10135 ± 553	9435	<0·0001
			InDel	4/38	4002 ± 255·9	3821	0·9030 / <0·0001
6c / S4d	Interburst spike count, DIV 28, MEA	Kruskal-Wallis	Ctrl	8/89	1496 ± 89·80	1439	
			G226R	4/65	2309 ± 118·2	2220	<0·0001
			InDel	4/38	1035 ± 57·24	1031	0·0055 / <0·0001
6c / S4d	NB/min, DIV 28, MEA	Kruskal-Wallis	Ctrl	8/89	4·80 ± 0·37	4·00	
			G226R	4/65	16·22 ± 1·44	13·60	<0·0001
			InDel	4/38	8·93 ± 1·07	7·50	0·0036 / 0·0014
6d	NB duration, DIV 28, MEA	Kruskal-Wallis	Ctrl	8/89	0·62 ± 0·02	0·59	
			G226R	4/65	1·16 ± 0·09	1·05	<0·0001
			InDel	4/38	0·88 ± 0·09	0·68	0·0489 / 0·1689
6d	Intra-NB spike count, DIV 28, MEA	Kruskal-Wallis	Ctrl	8/89	1117 ± 49·05	1122	
			G226R	4/65	1831 ± 180·80	1329	0·1249
			InDel	4/38	1579 ± 256·80	850	>0·9999 / 0·0606

6d	NB spike frequency, DIV 28, MEA	Kruskal-Wallis	Ctrl	8/89	148·1 ± 3·28	150·1	
			G226R	4/65	115·9 ± 3·49	116	<0·0001
			InDel	4/38	127·2 ± 8·49	111·2	0·0001 / >0·9999
S2a	Normalized charge, 10Hz, autaptic	Permutation-based mixed-design ANOVA/ Posthoc Permutation ind. ttest	Ctrl	4/35			
			G226R	4/29			0·8333
			InDel	4/26			0·9985
S2a	Normalized charge, 20Hz, autaptic	Permutation-based mixed-design ANOVA/ Posthoc Permutation ind. ttest	Ctrl	4/31			
			G226R	4/28			0·1659
			InDel	4/25			0·2996
S2b	PP ratio,<br><br>50 ms, autaptic	Kruskal-Wallis	Ctrl	4/31	0·91 ± 0·05	0·94	
			G226R	4/29	0·84 ± 0·04	0·84	0·6709
			InDel	4/25	0·81 ± 0·04	0·82	0·4544

S2b	PP ratio, 100 ms, autaptic	Kruskal-Wallis	Ctrl	4/35	0·92 ± 0·04	0·89	
			G226R	4/30	0·92 ± 0·03	0·91	>0·9999
			InDel	4/26	0·89 ± 0·03	0·89	>0·9999
S2b	PP ratio, 200 ms, autaptic	Kruskal-Wallis	Ctrl	4/37	0·97 ± 0·06	0·89	
			G226R	4/37	0·88 ± 0·05	0·87	0·8538
			InDel	4/36	0·84 ± 0·04	0·83	0·1723
S2b	PP ratio, 1000 ms, autaptic	Kruskal-Wallis	Ctrl	4/37	0·90 ± 0·04	0·91	
			G226R	4/34	0·86 ± 0·03	0·87	0·6893
			InDel	4/33	0·88 ± 0·04	0·90	>0·9999
S3c	Bassoon/Homer colocalzation, network	Kruskal-Wallis	Ctrl	4/27	130 ± 16·79	115	
			G226R	4/35	89·82 ± 14·15	56	0·0879
			InDel	4/33	179·72 ± 18·68	166	0·1548
S3c	Dendritic length, network	Kruskal-Wallis	Ctrl	4/27	4·58 ± 0·83	3·29	
			G226R	4/35	3·13 ± 0·35	2·61	0·2878

			InDel	4/33	2·89 ± 0·46	2·22	0·0428
S3c	Synaptic density, network	Kruskal-Wallis	Ctrl	4/27	4·68 ± 0·82	3·37	
			G226R	4/35	3·67 ± 0·61	2·38	0·6158
			InDel	4/33	10·95 ± 1·98	7·38	0·0041
S4a	Capacitance, network	Kruskal-Wallis	Ctrl	4/24	46·30 ± 4·12	39·57	
			G226R	4/25	57·58 ± 3·89	55·74	0·1262
			InDel	3/13	33·85 ± 2·41	34·62	0·1179
S4a	AP threshold, network	Kruskal-Wallis	Ctrl	4/23	-25·71 ± 1·90	-26·25	
			G226R	4/24	-28·13 ± 1·55	-29·60	>0·9999
			InDel	3/12	-25·99 ± 2·09	-27·77	>0·9999
S4a	AP amplitude, network	Kruskal-Wallis	Ctrl	4/24	79·91 ± 3·83	83·92	
			G226R	4/25	80·68 ± 2·24	79·96	>0·9999
			InDel	3/13	73·81 ± 3·79	70·80	0·3553
S4a	AP halfwidth, network	Kruskal-Wallis	Ctrl	4/24	1·64 ± 0·07	1·60	

			G226R	4/24	1·64 ± 0·06	1·59	>0·9999
			InDel	3/13	1·71 ± 0·06	1·70	>0·9999
S4a	Afterhyperpolarization, network	Kruskal-Wallis	Ctrl	4/23	-45·17 ± 1·25	-45·78	
			G226R	4/24	-48·83 ± 0·97	-48·83	0·1541
			InDel	3/12	-50·71 ± 1·30	-50·96	0·0243
S4c / S4d	Bursts/min, DIV 28, MEA	Kruskal-Wallis	Ctrl	8/89	6·11 ± 0·48	5·18	
			G226R	4/65	22·47 ± 1·47	18·20	<0·0001
			InDel	4/38	10·14 ± 1·10	9·22	0·0144 / <0·0001
S4c	Burst duration, DIV 28, MEA	Kruskal-Wallis	Ctrl	8/89	0·35 ± 0·01	0·36	
			G226R	4/65	0·55 ± 0·04	0·48	0·0017
			InDel	4/38	0·55 ± 0·07	0·34	>0·9999 / 0·1462
S4c	Intraburst spike count, DIV 28, MEA	Kruskal-Wallis	Ctrl	8/89	71·64 ± 2·56	72·35	
			G226R	4/65	94·71 ± 8·61	81·09	>0·9999
			InDel	4/38	108·70 ± 19·39	53·93	0·8186 / 0·2641

S4c	Burst spike frequency, DIV 28, MEA	Kruskal-Wallis	Ctrl	8/89	182·9 ± 2·49	186·3	
			G226R	4/65	138·1 ± 2·47	138·5	<0·0001
			InDel	4/38	152·8 ± 6·87	136·1	<0·0001 / 0·2551
S4d	Total spike count, DIV 35, MEA	Kruskal-Wallis	Ctrl	8/62	5831 ± 725·2	3865	
			G226R	4/63	13893 ± 1102	12189	<0·0001
			InDel	4/39	6945 ± 373·2	6563	0·0827
S4d	Total spike count, DIV 42, MEA	Kruskal-Wallis	Ctrl	8/62	3978 ± 385·8	3188	
			G226R	4/36	13150 ± 1669	10626	<0·0001
			InDel	4/34	7962 ± 736·9	8163	0·0001
S4d	Interburst spike count, DIV 35, MEA	Kruskal-Wallis	Ctrl	8/62	1798 ± 175·5	1278	
			G226R	4/63	3056 ± 184	3185	<0·0001
			InDel	4/39	1369 ± 76·02	1380	>0·9999
S4d	Interburst spike count, DIV 42, MEA	Kruskal-Wallis	Ctrl	8/62	1174 ± 79·91	1076	
			G226R	4/36	3292 ± 276·2	3475	<0·0001

			InDel	4/34	1151 ± 113·8	1113	>0·9999
S4d	NB/min, DIV 35, MEA	Kruskal-Wallis	Ctrl	8/62	7·18 ± 0·96	5·60	
			G226R	4/63	28·43 ± 3·97	15·00	<0·0001
			InDel	4/39	12·09 ± 1·51	9·60	0·00599
S4d	NB/min, DIV 42, MEA	Kruskal-Wallis	Ctrl	8/62	8·97 ± 1·15	5·30	
			G226R	4/36	22·39 ± 4·59	10·60	0·0055
			InDel	4/34	14·61 ± 1·95	13·00	0·0272
S4d	Bursts/min, DIV 35, MEA	Kruskal-Wallis	Ctrl	8/62	9·18 ± 1·12	7·50	
			G226R	4/63	31·98 ± 4·08	16·95	<0·0001
			InDel	4/39	14·65 ± 1·55	12·37	0·0063
S4d	Bursts/min, DIV 42, MEA	Kruskal-Wallis	Ctrl	8/62	10·06 ± 1·19	7·13	
			G226R	4/36	24·84 ± 4·59	11·77	0·0015
			InDel	4/34	16·90 ± 1·99	16·43	0·0118

Supplementary References

1. Colbert KN, Hattendorf DA, Weiss TM, Burkhardt P, Fasshauer D, Weis WI. Syntaxin1a variants lacking an N-peptide or bearing the LE mutation bind to Munc18a in a closed conformation. Proc Natl Acad Sci. 2013 Jul 30;110(31):12637–42. doi:10.1073/pnas.1303753110

2. Zhou Q, Lai Y, Bacaj T, Zhao M, Lyubimov AY, Uervirojnangkoorn M, et al. Architecture of the synaptotagmin–SNARE machinery for neuronal exocytosis. Nature. 2015 Sep 3;525(7567):62–7. doi:10.1038/nature14975

3. Sehnal D, Bittrich S, Deshpande M, Svobodová R, Berka K, Bazgier V, et al. Mol* Viewer: modern web app for 3D visualization and analysis of large biomolecular structures. Nucleic Acids Res. 2021 Jul 2;49(W1):W431–7. doi:10.1093/nar/gkab314

4. Berman HM, Westbrook J, Feng Z, Gilliland G, Bhat TN, Weissig H, et al. The Protein Data Bank. Nucleic Acids Res. 2000;28(1):235–42. doi:10.1093/nar/28.1.235.
